# Genomic DNA methylation profile in peripheral blood of children with congenital biliary dilatation

**DOI:** 10.1186/s12920-025-02223-3

**Published:** 2025-10-23

**Authors:** Chenyao Wang, Yuelan Zheng, Yan Wang, Qi Feng

**Affiliations:** Department of Hepatobiliary Tumor Surgery, Shen Zhen Children’s Hospital, Shenzhen, Guangdong China

**Keywords:** Congenital biliary dilatation, DNA methylation, Epigenetics, Differentially methylated regions (DMRs)

## Abstract

**Background:**

Congenital biliary dilatation (CBD) is a prevalent congenital biliary disease in children, particularly in Asian populations, yet its etiology remains poorly understood. This study aimed to investigate the genome-wide DNA methylation profile in the peripheral blood of children with CBD to identify potential epigenetic mechanisms involved in its pathogenesis.

**Methods:**

Genome-wide DNA methylation profiles were compared between whole blood samples from 37 children with CBD and 24 healthy controls using the Illumina Infinium Human MethylationEPIC (850 K) BeadChip. Bioinformatic analyses were performed to identify differentially methylated positions (DMPs) and regions (DMRs), and functional enrichment analysis was conducted on associated genes.

**Results:**

We identified 24,230 differentially methylated sites associated with 5,863 genes. Among these, 8,313 sites were hypermethylated and 15,917 were hypomethylated in CBD patients compared to controls. 54 significant differentially methylated regions (DMRs) were also detected. Functional enrichment analysis revealed that the differentially methylated genes were significantly enriched in key biological pathways, most notably the T cell receptor signaling pathway.

**Conclusion:**

This study presents the first comprehensive analysis of genome-wide DNA methylation in CBD, revealing significant epigenetic alterations in peripheral blood. These findings suggest that aberrant DNA methylation, particularly in genes regulating immune pathways, may play a critical role in the development of CBD and could provide valuable insights for identifying novel diagnostic biomarkers.

**Supplementary Information:**

The online version contains supplementary material available at 10.1186/s12920-025-02223-3.

## Background

Congenital biliary dilatation (CBD) is a relatively rare biliary tract disease [1]. In contrast to the incidence observed in Europeans and Americans, the incidence of CBD is much higher in Asia,particularly among Asian populations,where it can be as high as 1/1, 000 [2]. The current reason for the high incidence among Asians remains unclear. CBD mainly affects infants and children, with a higher proportion in females, and the incidence ratio of male to female is about 1:4 [3]. The widely recognized pathogenesis involves congenital biliary mal junction. The dilated bile ducts cause dilatation-stenosis-like biliary stricture, which subsequently leads to the abnormality of bile dynamics, causing cholestasis and then inducing biliary tract inflammation [4, 5]. At present, the generally accepted surgical method is complete cyst resection and hepatic duct jejunal anastomosis [6]. However, it may increase life-threatening risks when undergoing operation at childhood [7]. Currently, the lack of effective biomarkers for early diagnosis of CBD greatly limits the treatment of CBD [8]. Therefore, screening effective biomarkers is crucial for the early diagnosis.

DNA methylation represents a significant epigenetic modification that can influence the expression and alternative splicing of genes, thereby maintaining genome stability [9, 10]. Nowadays, many researchers have reported that aberrant DNA methylation plays a critical role in the occurrence and development of a range of congenital diseases by regulating the abnormally methylated loci in patients [11, 12]. Nevertheless, there is a paucity of research investigating the occurrence of aberrant DNA methylation in patients with CBD. Mori H et al. [8] demonstrated that Biliary tract cancer may develop later in children with CBD and adults with maljunction, potentially mediated through histone deacetylase (HDAC) and activation-induced cytidine deaminase (AID) expression via epigenetic and genetic mecchanisms [8]. Wanvisa Udomsinprasert et al. [13] demonstrated a positive correlation between Alu and LINE-1 methylation and relative telomere length in patients with biliary atresia (BA), indicating that retrotransposon hypomethylation is associated with plasma 8-OHdG in BA patients. However, the role of DNA methylation in the underlying mechanisms of CBD remains unclear.

In this study, we first evaluated the genome-wide DNA methylation characteristics of CBD through the Illumina Infinium Human Methylation 850 k BeadChip.To investigate the DNA methylation patterns in children with CBD and a control group, we performed a comparative analysis of the genome-wide DNA methylation profiles of whole blood samples from patients with CBD and a control group. Moreover, this is the first study to investigate genome-wide epigenetic landscapes of congenital bile duct dilatation in children, thereby elucidating the potential epigenetic differences associated with CBD.

## Methods

### Study objectives

Thi case–control study recruited consecutive patients with CBD diagnosed at the Shenzhen Children's Hospital, between March 2020 and March. In norderto be eligible for inclusion in the study, participants had to meet the following criteria: a confirmed diagnosis of CBD, as determined by computed tomography (CT) and/or magnetic resonance imaging (MRI), and the provision of informed consent in writing. The control subjects exhibited no symptoms or history of hepatobiliary disease. Finally, 37 patients and 24 controls were enrolled in the study following the procurement of written informed consent from all subjects. All research protocols were approved by the Ethics Committee of Shen Zhen Children’s Hospital (No.202002902) according to the State Drug Administration and the National Health and Wellness Commission's Code for Quality Management of Drug Clinical Trials (2020),Code for Quality Management of Clinical Trials of Medical Devices (2016), the Ministry of Health's "Biomedical Research Involving HumansEthical Review Measures (2016), the Declaration of Helsinki, and the CIOMS Ethical Principles for Medical Research Involving Human Subjects.

Gender, age, systolic blood pressure (SBP), diastolic blood pressure (DBP), Triglycerides (TG), Total Cho­lesterol (TC), Alanine aminotransferase (ALT), total bilirubin(TBil) and γ-glutamyl transferase (GGT) were all recorded at the time of admission. 

### Illumina Infinium Human Methylation 850 K bead chip

DNA from whole blood of patients and controls was meticulously extracted using a reagent kit sourced from Yeasen(China), in strict compliance with the manufacturer's protocol. The DNA samples' purity and yield were determined using a Thermo Scientific NanoDrop 2000, ensuring the quality for subsequent analysis. For the conversion to bisulfite-modified DNA, 500 ng of each genomic DNA sample was processed with the EZ DNA Methylation-Gold Kit (Zymo Research, USA), following the manufacturer's instructions. Employing the Illumina Human Methylation 850 K BeadChip (LC Sciences, Hangzhou, China), we conducted a thorough analysis of methylation patterns. The platform's probes and hybridization conditions were tailored to the bisulfite-modified sequences, with the resultant fluorescence intensity ratios providing a direct measure of methylation status. Our dataset has been uploaded to the NCBI Gene Expression Omnibus (GEO) database. The project accession number is GSE275555, and the data can be accessed at the following link: https://www.ncbi.nlm.nih.gov/geo/query/acc.cgi?acc=GSE275555.

### DNA methylation quality control and processing

The IDAT files from the array data were processed using the ChAMP package (version 3.20) in R (version 3.3.3). Samples were excluded if they had a Failed CpG Fraction exceeding 0.10. Probes were excluded based on a *p-*value threshold of 0.01, mapping ambiguity, non-CpG status, or insufficient bead counts in over 5% of the samples. The raw data are available in Additional file 1:Table S1. Methylation status for all probes was quantified as Δβ values, calculated as the ratio of methylated probe intensity to the total probe intensity. A probe was classified as hypermethylated if its mean Δβ value in the CBD cases was greater than the control mean by at least 0.2, and as hypomethylated if its mean Δβ value in the CBD cases was less than the control mean by at least −0.2.

### Bioinformatic analysis

Microarray raw data from the Illumina Infinium Human Methylation 850 K BeadChip were subjected to a comprehensive pre-processing workflow using the R minfi package [14] (Version 1.42.0). This workflow included filtering, value correction, and normalization to address background noise, technical variations, and probe reliability, respectively.The R IMA package [15] (Version 3.1.2) facilitated the detection of methylation differences across sample groups. Differential methylation was assessed using beta values, which scale from 0 to 1, with higher values indicating increased methylation.The beta-mixture quantile dilatation (BMIQ) [16]method corrected for probe design and intra-array variability. Inter-array batch effects were mitigated using the ComBat function. Subsequent differential methylation analysis was conducted using the limma-based ChAMP package (Version 3.20) [15]. This analysis identified differentially methylated positions (DMPs) and regions (DMRs) by comparing beta values across sample groups, with adjustments for probe design, intra-array variability, and batch effects.DMPs were defined by a |Δβ| threshold of ≥ 0.20 and a Holm-adjusted *p* value ≤ 0.05, while identified as clusters of probes within a 2000 bp window, exhibiting consistent methylation changes and meeting the same statistical significance criteria [17]. The Probe Lasso method was applied for DMR analysis.Functional annotation of genes within DMRs was conducted using the Kobas tool [18] (Version 3.0) (http://kobas.cbi.pku.edu.cn/kobas3), accessible at Kobas. Enrichment analysis for Gene Ontology and Kyoto Encyclopedia of Genes and Genomes (KEGG) pathways was performed, with a false discovery rate < 0.05 deemed significant.

### Pyrosequencing analysis

DNA was subjected to bisulfite conversion using the EZ DNA Methylation-Gold Kit (ZYMO Research) following the manufacturer's guidelines, post-extraction. The converted DNA was then amplified via PCR with the TaKaRa EpiTaq HS Kit (TaKaRa) in a 50 μL reaction, incorporating 20 pmol/L sequencing primer and 50 ng of bisulfite-converted DNA. PCR products, after agarose Gel purification, were pyrosequenced with the PyroMark Gold Q96 system (Qiagen), according to the provided protocol. PyroMark Q96 software (v2.5.8, Qiagen) was utilized for data acquisition and analysis.

### Quantitative real‐time PCR

For RNA analysis, total RNA was isolated using TRIzol reagent, and cDNA was synthesized with the Promega GoScript Reverse Transcription Mix. mRNA levels were assessed via quantitative real-time PCR (qRT-PCR) employing the GoTaq qPCR Master Mix (Promega) on an ABI StepOnePlus system. The 2^-ΔΔCt method was applied to determine relative gene expression levels, with experiments conducted in triplicate. Primer sequences are detailed in Additional file 1:Table S2.

### Statistical analysis

To investigate the phenotypic differences between the CBD patients and healthy controls, we utilized a series of statistical tests. Continuous variables were compared using the Student's t-test, assuming normal distribution, while the Mann–Whitney U test was employed for non-normally distributed data. Categorical variables were analyzed using the Chi-square test. A two-tailed *p-*value of less than 0.05 was considered statistically significant. All statistical analyses were conducted using Graphpad prism8.0.

## Results

### Blood genomic DNA methylation variations in CBD patients

In this study, we enrolled 61 individuals, including 37 children diagnosed with Congenital Biliary Dilatation (CBD) (aged 40.28 ± 4.506 months) and 24 age-and sex-matched healthy controls(aged 40.10 ± 6.220 months). A comprehensive set of demographic, clinical, and laboratory data for these participants is presented in Table 1. There were no significant differences in age or gender between CBD patients and healthy controls. However, CBD patients had significantly higher levels of liver enzymes, such as ALT (*P* < 0.05), AST (*P* < 0.001) and TBil (*P* < 0.001).

**Table 1:** Characteristics of cases and controls

	CBD(*n* = 37)	Healthy controls(*n* = 24)	*P*
Age(months)	40.28 ± 4.506	40.10 ± 6.220	*P* = 0.8962
Sex(M/F)	18/19	11/13	*P* > 0.9999
Alanine aminotransferase (U/L)	27.97 ± 16.86	18.210 ± 9.51	*P* = 0.0125^*^
Aspartate aminotransferase (U/L)	61.42 ± 8.997	28.57 ± 11.543	*P* < 0.0001
Total bilirubin (μmol/L)	20.78 ± 9.230	8.732 ± 2.999	*P* < 0.0001

Data are presented as mean ± SD or number. *P* < 0.05 CBD vs. Healthy controls

**Fig. 1:**
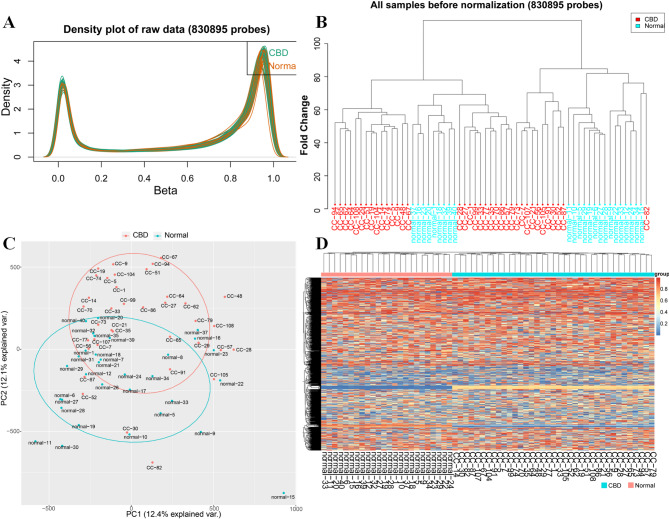
Density plot and principal component analysis (PCA) plot. **A** Density plot. This plot illustrates the distribution of β values representing methylation levels for the 37 CBD patients (depicted in green) and the 27 healthy controls (depicted in orange. **B** Raw Sample Cluster plot before normalization (830,895 probes). **C** PCA plot based on all CpG sites. No discernable separation is observed between the CBD patients (red dots) and the healthy controls (blue dots). PC1 explains 12.4% variance, and PC2 explains 12.1% variance. **D** Heatmap displaying methylation differences across 24,230 DMPs. Each row represents a DMP, and each column represents a subject. Red indicates hypermethylation in CBD patients compared to controls, while blue indicates hypomethylation

We performed a genome-wide DNA methylation analysis using the Illumina Infinium Human Methylation 850 k BeadChip, analyzing 830,895 CpG sites. After quality control and normalization, methylation levels were compared between 37 CBD cases and 24 controls, applying a stringent threshold of adjPVal < 0.05 (BH-adjusted) and |Δβ|> 0.10. The density plot indicated that the methylation levels in the samples from patients with CBD and healthy controls were relatively similar (Fig. 1A, B). Differentially methylated sites are reported with β-values.Principal component analysis (PCA) on the 832,347 CpG sites did not did not fully separate CBD patients from controls (Fig. 1C). The analysis identified 24,230 differentially methylated sites(adj *P* value < 0.05),which were mapped to 5,863 genes (Fig. 1D).

### CBD-associated site-specific differences in DNA methylation

**Fig. 2:**
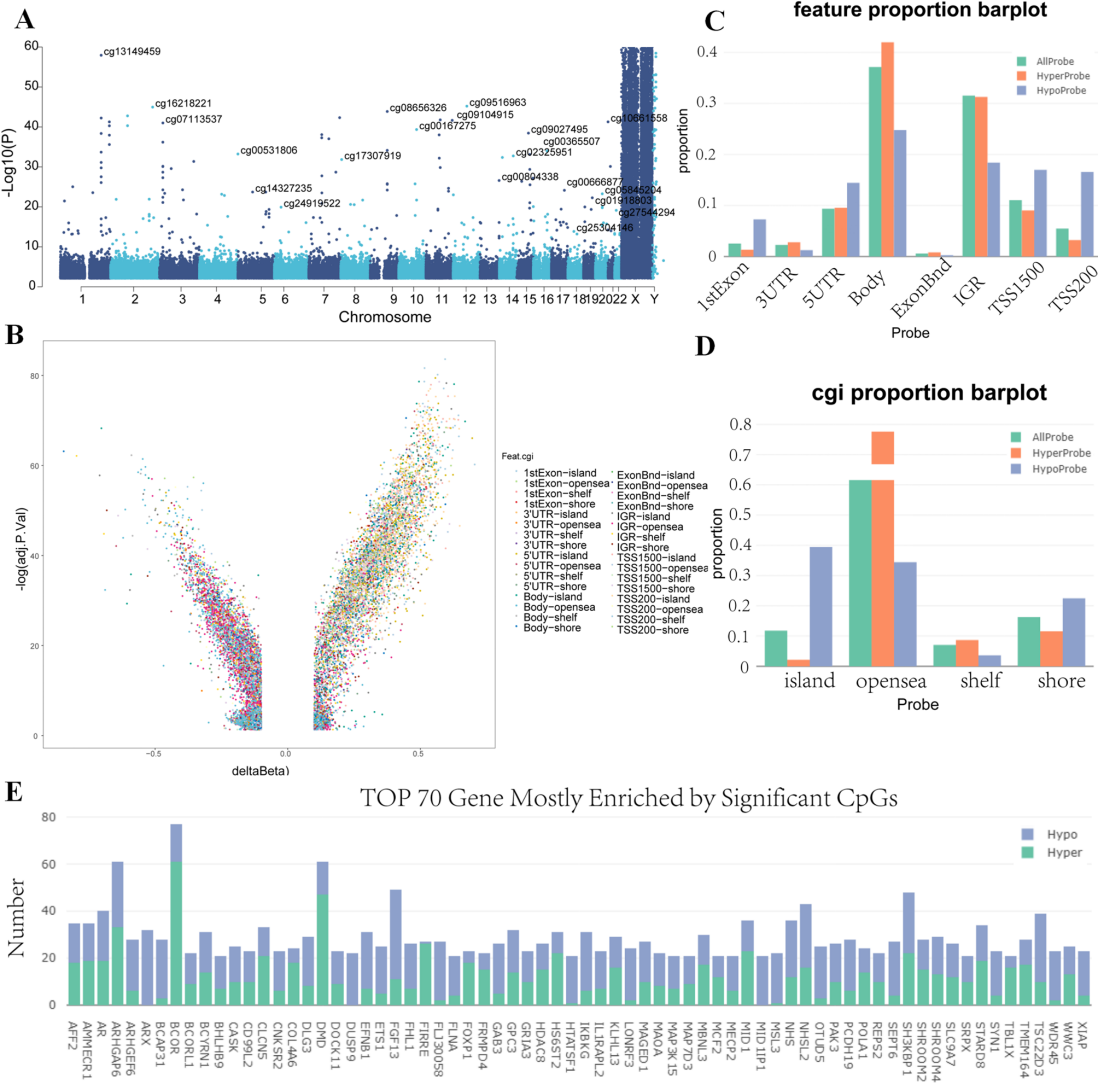
Differentially methylated positions (DMP) analysis in Congenital biliary dilatation (CBD) cases and controls in whole blood. **A** Manhattan plot of top differentially methylated positions (DMPs) in CBD versus control. **B** Volcano plot of top DMPs and position of methylation probes in relation to the gene (TSS, transcription start site;IGR, intergenic region; UTR, untranslated region). **C **Genomic features of differentially methylated sites in CBD. The distribution of hypomethylation and hypermethylation sites is compared with all probes on the Illumina array. **D** Graph showing percentages of differentially methylated sites according to their CpG content. **E** Top 70 genes with the most enriched significantly differentially methylated sites

This study presents a comparative epigenomic analysis between CBD patients and controls,focusing on DNA methylation differences. Utilizing the Illumina Human Methylation 850 K Beadchip, we identified 24,230 differentially methylated positions (DMPs) with statistically significant methylation variance in CBD cases(corrected *P* < 0.05). Figure 2A displays a Manhattan plot of the top DMPs associated with CBD. Of these, 8,313 (34.31%) showed hypermethylation (adjPVal < 0.05, Δβ > 0.10), and 15,917 (65.69%) displayed hypomethylation(adjPVal < 0.05, Δβ < 0.10) (Fig. 2B).The results indicate that the majority of the significantly differentially methylated sites in children with CBD were identified within the gene body area. Furthermore, the distribution of hypomethylation and hypermethylation sites in comparison to all probes on the 850 k Illumina array revealed no significant difference (Fig. 2C and D). The 24,230 DMPs with annotated biological functions were mapped to 5,863 distinct genes (Additional file 1:Table S3). Figure 2E illustrates the top 70 genes with the most pronounced differential methylation.

**Fig. 3:**
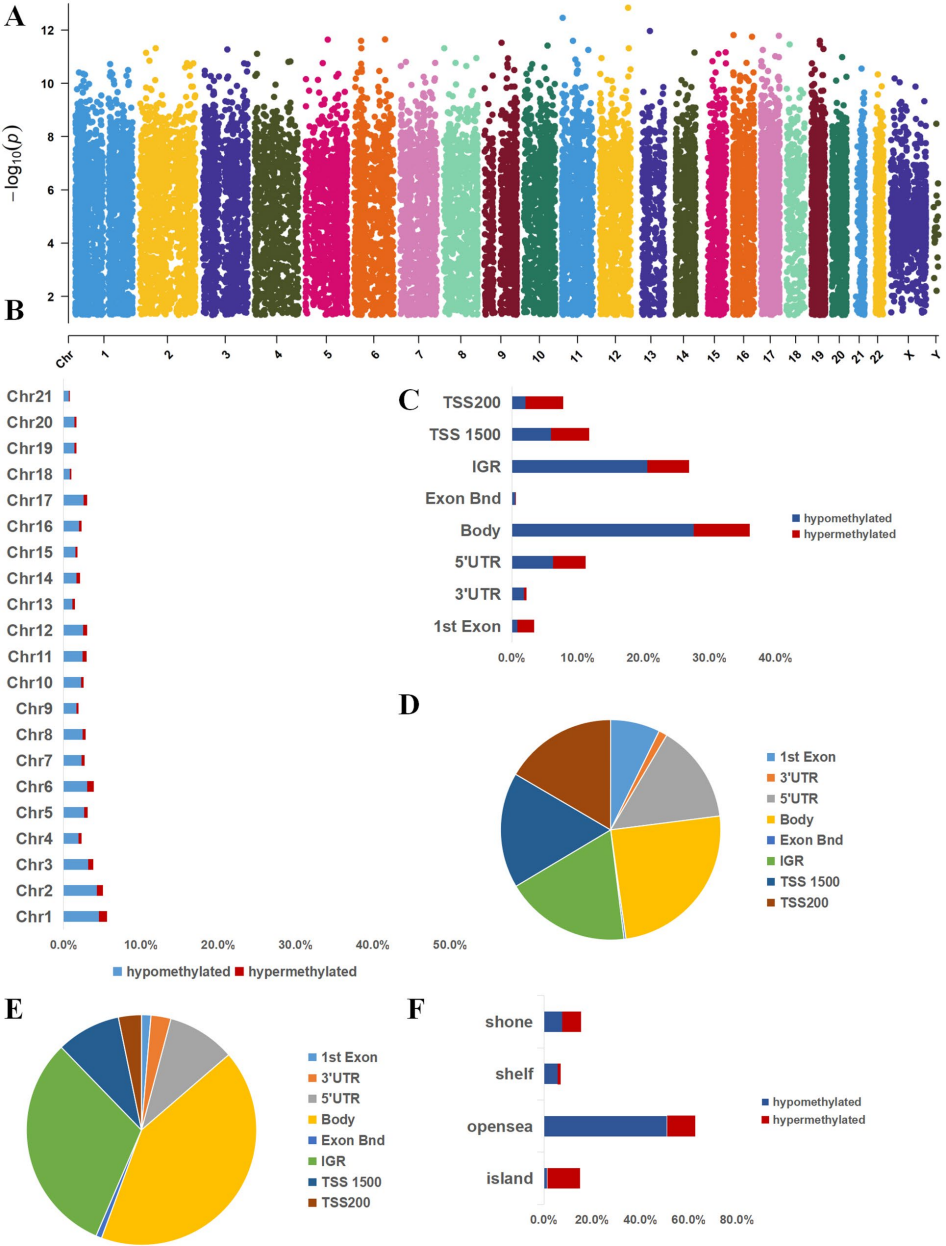
Distribution of methylation sites in the chromosomes and functional analysis of DMPs. **A** Distribution of 24,230 DMPs across chromosomes, with hypermethylation in red and hypomethylation in blue. **B** Distribution of DMPs along the chromosome, showing the percentage of hypermethylated (red) and hypomethylated (blue) sites. **C** The distribution percentages of the 24,230 DMPs across different gene regions,including gene body,TSS1500, TSS200, 3’UTR, 5’UTR, 1 st Exon and intergenic regions (IGR). Red bars represent hypermethylated DMPs, whereas blue bars represent hypomethylated DMPs. **D** The distribution of hypermethylation sites. **E** The distribution of hypomethylation sites. **F** The distribution percentages of the 439 DMPs in different CpG island regions,including Shore, Shelf, CpG island and Open Sea

The differentially methylated sites were distributed across all chromosomes (Fig. 3A and B). A detailed examination of hypermethylated sites revealed the following distribution across functional genomic domains: 1,529 (18.39%) in noncoding regions, 2,789 (33.55%) at transcription start sites, 2,061 (24.79%) in coding regions and 104 (1.25%) within the 3' untranslated region (UTR) termini (Fig. 3C and D). Hypomethylated sites were distributed as follows: 4981(31.29%) in noncoding intergenic regions, 6679(41.96%) in coding regions, 1950(12.25%) at transcription start sites, and 446 (2.8%) were in the end of a 3' UTR (Fig. 3C and E)). Among methylation sites in promoter regions, 3624 were located in a CpG island, 3709 were in a shore, 15,211 were in an opensea, 1686 in shelf (Fig. 3F).

### Ontology functional analysis of differentially methylated genes in the blood of patients with CBD

**Fig. 4:**
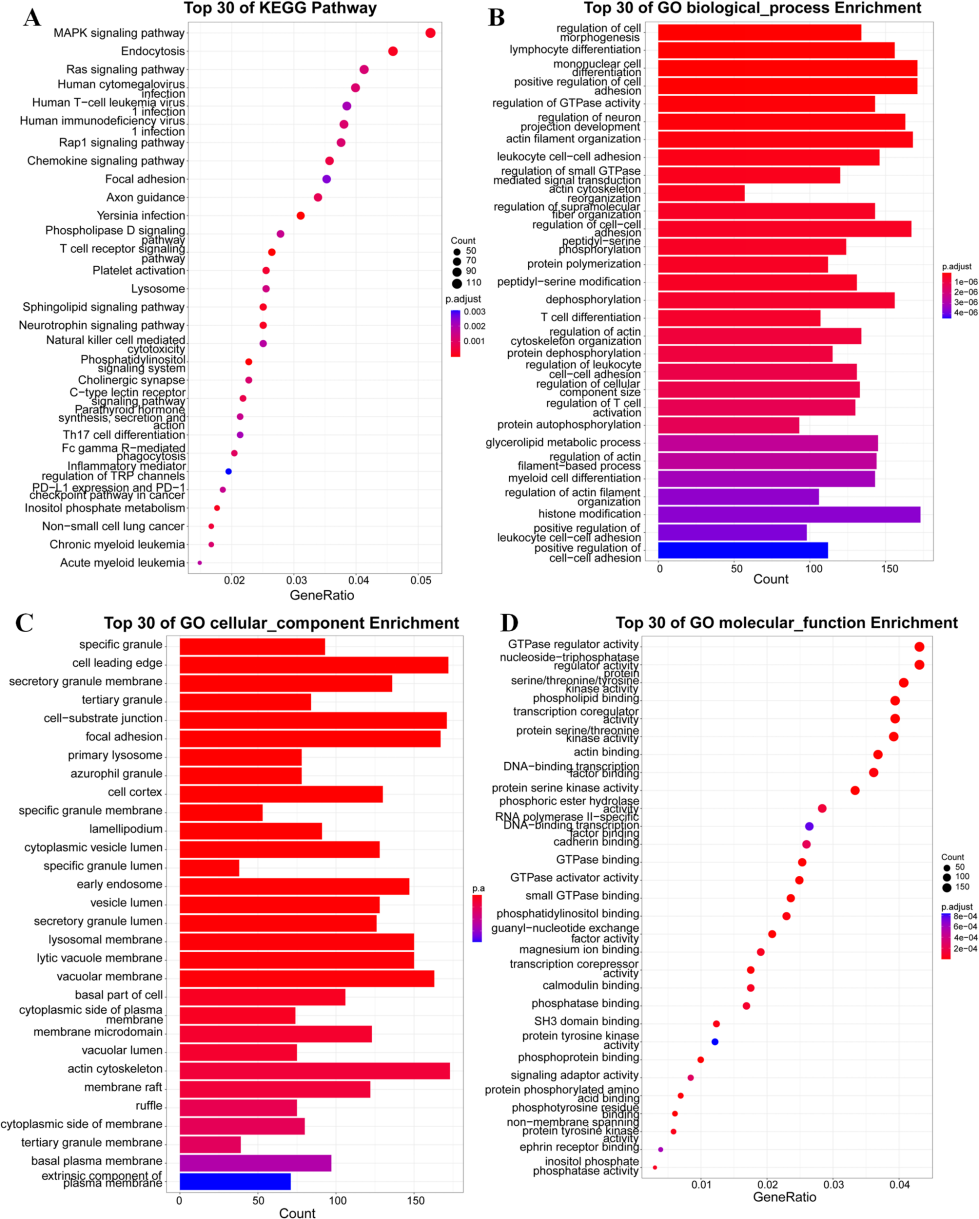
Ontological and Functional Enrichment Analysis of Differentially Methylated Genes. **A** Top 30 KEGG pathway enrichment. **B** Top 30 Gene Ontology Biological Process Enrichment. **C** Top 30 Gene Ontology Cellular Component Enrichment. **D** Top 30 Gene Ontology Molecular Function Enrichment.The vertical axis indicates the name of the KEGG pathway or GO category, the horizontal axis represents the enrichment factor, and the size of the circle indicates the number of differential methylation-related genes involved in the pathway or category

The dataset encompassing 24,230 biologically annotated sites was mapped to 5,863 distinct genes. A gene biological process analysis(Bonferroni corrected *p-*value < 0.05) revealed significant enrichment of these differentially methylated genes (DMGs) in processes such as metabolic regulation, cell signaling, and the catabolism and metabolism of cellular and intracellular compounds, as well as the regulation of stimuli from both endogenous and exogenous sources (Fig. 4A, B). Cellular component analysis (Bonferroni corrected *p-*value < 0.05) indicated a notable enrichment of these genes within intracellular compartments, particularly at cell-substrate junctions and the leading edges of cells (Fig. 4C). Molecular function analysis (Bonferroni corrected *p-*value < 0.05) of DMGs showed significantly enrichment in the T cell receptor signaling pathway, Phosphatidylinositol signaling system, Inositol phosphate metabolism and Endocytosis (Fig. 4D). The gene ontology enrichment analyses, focusing on the three aforementioned categories, highlighted the top 10 most significant *p-*values for each.

### Functional analysis of DMRs of differentially methylated genes in the blood of patients with CBD

**Fig. 5:**
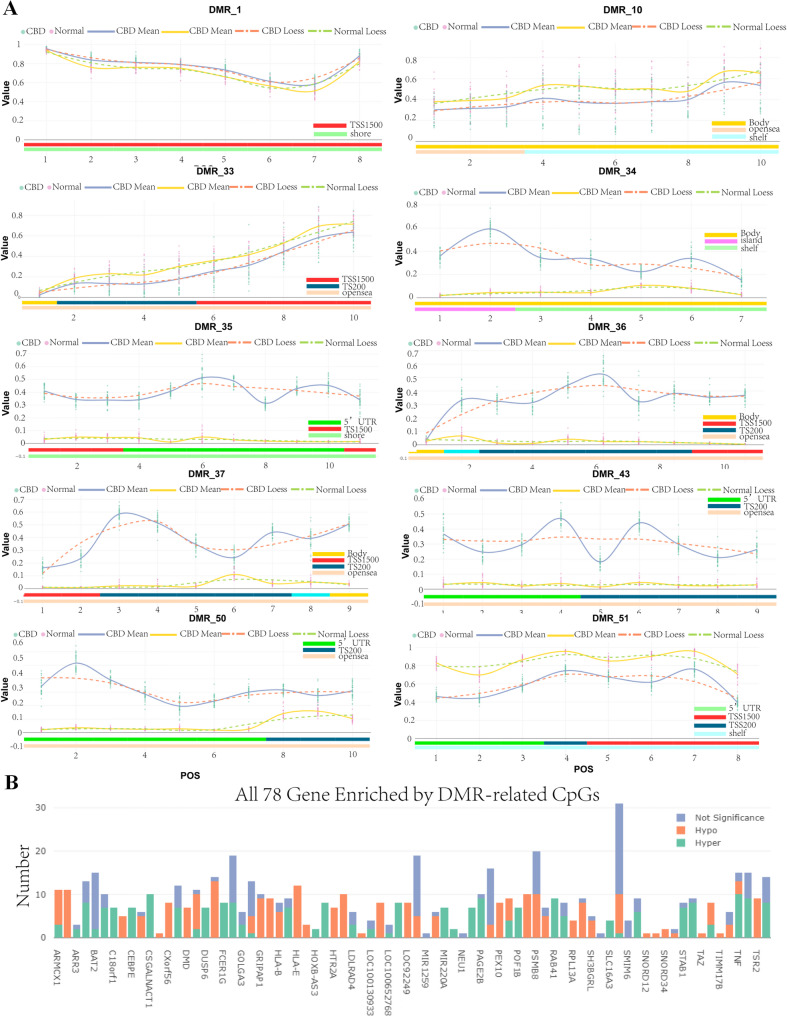
DMR (differentially methylated region) analysis. **A** Differentially methylated regions(DMR_1, DMR_34, DMR_35, DMR_36, DMR_37,DMR_43 and DMR_50) demonstrate that the expression of these genes in CBD cases is apparently higher than in the control group; DMR_10, DMR_33 and DMR_51 are hypomethylated between CBD cases and the control group. (method = "ProbeLasso"; minProbes = 7;adjPvalDmr < 0.05 (adjust.method = "BH")mean LassoRadius = 375; minDmrSep = 1000, minDmrSize = 50, adjPvalProbe < 0.05.). **B** The bar chart shows that TOP 78 hyper-enriched gene by DMR-related CpGs among all 5,863 genes

According to the differential methylation region (DMR) analysis, we found that 54DMR between CBD cases and the control group showed a significant difference(DMR_1 ~ DMR_54, Table 2, Additional file 1:Table S4). As is shown in Fig. 5A and Table 2, DMR_1, DMR_34-37, DMR_43 and DMR_50 were hypermethylated between CBD cases and the control group, while DMR_10, DMR_33 and DMR_51 were hypomethylated. Notably, DMR_1 in the PEX10 gene demonstrate a marked difference, with higher methylation values in the CBD group.Among the differentially methylated positions sites,cg13306335,cg14373988,cg20136951,cg20823695,cg24439334,cg20664247,cg23626733 and cg23629166 was hypermethylated in TSS1500 and shore region (Fig. 5A). DMDCSGALNACT1 gene, present in DMR_33, had DMPs cg15379858, cg11155735, cg23328404, cg02694058, cg11505841, cg14818701, cg18325192, cg20450123, cg14854503 and cg24423468 hypomethylated in the transcription start site and opensea region. In total, DMR_1 to DMR_54 encompassed 489 differentially methylated positions related to 78 genes (Fig. 5B).

**Table 2:** Top 30 Differentially methylated region (DMR) between CBD cases and the control group

DMR	Gene	dmrpRank	CHR	dmrSize	dmrCoreStart	dmrCoreEnd	dmrCoreSize
DMR_1	PEX10	23	1	174	2345333	2345475	143
DMR_6	DUSP6	28	12	316	89744471	89744701	231
DMR_7	GOLGA3	41	12	3518	133343405	133346306	2902
DMR_8	HTR2A	26	13	220	47472140	47472349	210
DMR_9	CEBPE	31	14	4192	23586582	23588616	2035
DMR_10	LOC404266;HOXB6	37	17	1864	46681111	46682319	1209
DMR_11	TMEM49;MIR21	33	17	4192	57915665	57918682	3018
DMR_12	LOC100130933;RECQL5	48	17	4192	73641809	73643683	1875
DMR_13	SLC16A3	46	17	3518	80196719	80199608	2890
DMR_14	C18orf1	27	18	967	13611370	13611824	455
DMR_15	FXYD1	54	19	3531	35629022	35630474	1453
DMR_16	RPL13AP5;SNORD33;SNORD35A;SNORD34;RPL13A;SNORD32A	35	19	2096	49993125	49994485	1361
DMR_17	SNORD12B;C20orf199;MIR1259;SNORD12	49	20	1917	47896448	47897451	1004
DMR_18	NFAM1	29	22	878	42828125	42828516	392
DMR_19	STAB1	32	3	878	52529085	52529524	440
DMR_2	NDUFS2;FCER1G	24	1	4250	161183762	161185092	1331
DMR_20	NA	25	6	604	28601271	28601519	249
DMR_21	TRIM39	52	6	2631	30296689	30298768	2080
DMR_22	HLA-E	19	6	2276	30459317	30460798	1482
DMR_23	DDR1	44	6	910	30860130	30860960	831
DMR_24	HLA-B	39	6	3518	31321433	31322996	1564
DMR_25	LTA;TNF	45	6	4192	31539973	31543686	3714
DMR_26	SNORA38;BAT2	53	6	1866	31590513	31592247	1735
DMR_27	VARS	43	6	525	31762409	31762901	493
DMR_28	NEU1;SLC44A4	50	6	2819	31832173	31834178	2006
DMR_29	ATF6B	38	6	1313	32086425	32087190	766
DMR_3	C10orf26	34	10	940	104535521	104536121	601
DMR_30	PBX2;GPSM3	47	6	5265	32157150	32161747	4598

### Pyrosequencing validation and qRT‐PCR validation

**Fig. 6:**
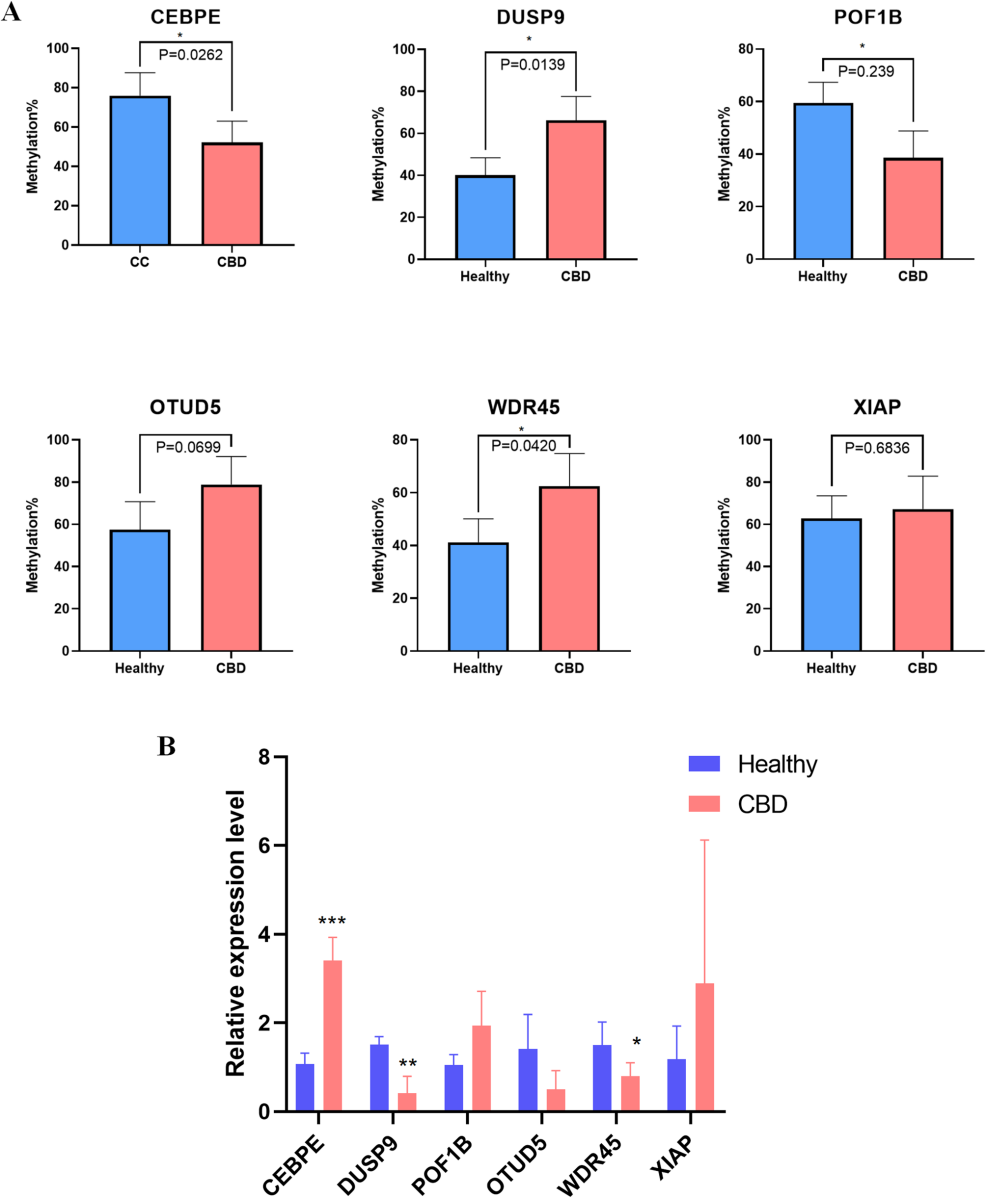
Validation of the results of the Illumina 850 K DNA methylation bead array analysis by pyrosequencing and qRT‐PCR. **A** Differential analysis between Illumina 850 K array data and pyrosequencing. This section presents the concordance between the methylation levels determined by the Illumina 850 K Bead Array and those verified by pyrosequencing for select genes: CEBPE, DUSP9, POF1B, OTUD5, WDR45, and XIAP. The correlation coefficients and their statistical significance are depicted, indicating the reliability of the array data. **B** Gene Expression Validation by Quantitative Real-Time PCR (qRT-PCR): The expression levels of the aforementioned genes are confirmed through qRT-PCR, with results normalized to the housekeeping gene β-actin to account for variations in sample*P < 0.05, **P < 0.01, ***P < 0.001

To explore the mechanisms underlying methylation alterations, we identified a set of genes involved in methylation regulation,MAP kinase inactivation, and inflammatory signaling pathways,including CEBPE, DUSP9, POF1B, OTUD5, WDR45, and XIAP. Six CpG sites within these genes showed the most significant methylation differences across the dataset. Methylation profiles of CpG sites in DUSP9, POF1B, and WDR45 varied markedly among the different groups (Fig. 6A).To further assess the functional relevance of these genes in CBD, we compared their mRNA expression levels between CBD patients and controls. Our results revealed significant expression divergences, in the expression of these genes between CBD patient samples and control samples (Fig. 6B). Our results revealed significant expression divergences, corroborating the methylation array data and suggesting a potential link between methylation status and gene expression in CBD pathophysiology.

## Discussion

Genome-wide DNA methylation is a critical and important mechanism in the field of epigenetics [13]. To our knowledge, this study is the first comprehensive analysis to reveal a distinct genome-wide DNA methylation profile in the whole blood of CBD patients. A total of 24,230 DMPs and 54 DMRs were identified between the CBD and control groups. The significantly enriched KEGG pathways of the DMPs included T cell receptor signaling pathway, Phosphatidylinositol signaling system, Inositol phosphate metabolism, Endocytosis and MAPK signaling pathway. GO analysis revealed DMPs were significantly enriched in the regulation of specific regulation of cell morphogenesis, lymphocyte differentiation, positive regulation of cell adhesion and regulation of GTPase activity. Among these 54 DMRs, 7 DMRs were hypermethylated and 3(DMR_10, DMR_33 and DMR_51)were hypomethylated in CBD cases. Pyrosequencing and qPCR validation confirmed the differential methylation and expression of key genes including CEBPE,DUSP9, POF1B and WDR45.Overall, our analysis suggests that changes in DNA methylation patterns might influence the development of CBD.

CCAAT/enhancer binding proteins (CEBPs) are significant transcription factors that regulate cell differentiaion and proliferation [19, 20]. Ewa Musialik et al. [21] reported high methylation levels at CEBPE promoter in AML patients, suggesting that DNA methylation of CEBPE plays a critical role in diseases. Dual-specificity phosphatase 9 (DUSP9) is a strong negative regulator of transcription factor activating MAPK pathways [22]. Jiho Choi et al. [23] demonstrated the epigenetic similarity of sex-matched Blastocyst-derived embryonic stem cells and gonad-derived embryonic germ cells and identify DUSP9 as a regulator of female-specific hypomethylation [23, 24]. Actin binding protein1 B(POF1B) is highly and specifically expressed in polarised epithelial tissues [25]. Valeria Padovano et al. [26] found that POF1B plays a key role in organizing epithelial monolayers by regulating the actin cytoskeleton [26]. Shinsuke Katsuno et al. [27] performed immunostaining of intramural nerves in gallbladders samples from CBD patients and observed hypoplasia of intramural vascularity and perivascular plexuses [27]. Therefore, we selected WD repeat domain phosphoinositide-interacting protein 4(WDR45) gene, which encodes a beta-propeller scaffold protein with a putative role in autophagy [28]. Pyrosequencing result show that WDR45 were hypermethylated in CBD patients. Based on the validation of candidate genes, Our study may provide useful information to explore the epigenetic pathology mechanism of the disease. Still, the relationship between these genes and CBD needs further research.

This study has several limitations.Despite the pioneering nature of our study in exploring the DNA methylation profiles in CBD, we are cognizant of its limitations, particularly the small sample size which restricts the generalizability of our findings. Our analysis indicates significant differential methylation in genes such as CEBPE, DUSP9, POF1B, and WDR45. Future research should aim to investigate the intricate relationships between methylation patterns, disease severity, and treatment outcomes to unravel the complex epigenetic mechanisms in CBD.Additionally, our study did not involve special handling or exclusion of sex chromosome data. This omission may lead to some bias in the interpretation of methylation levels, especially when considering mixed-gender samples. The potential for gender-related biases in the methylation patterns observed on sex chromosomes should be noted, and we recommend that future research considers the impact of sex chromosomes separately to better understand their role in CBD.

## Conclusion

In summary, this study provides the first genome-wide methylation landscape of congenital biliary dilatation in children. We identified widespread and significant alterations in DNA methylation patterns in the peripheral blood of CBD patients, implicating key genes and pathways, notably those involved in immune regulation like the T cell receptor signaling pathway. The successful validation of methylation and expression changes in genes such as CEBPE, DUSP9, POF1B, and WDR45 underscores their potential roles in CBD pathogenesis. Future studies with larger cohorts are warranted to confirm these findings and to explore the translational potential of these epigenetic markers for improving diagnosis and understanding the pathophysiology of CBD.

## Supplementary Information

Additional file 1: Table S1. Illumina Human Methylation 850 K Beadchip Raw Data. Table S2. Primers of this study. Table S3. CBD-associated site differences in DNA methylation. Table S4. Differentially methylated positions of DMR

Additional file 2

## Data Availability

All relevant data, including the original sequencing data, are accessible both within the manuscript and its Additional files. The genome-wide methylation microarray data generated in this study have been deposited in the NCBI Gene Expression Omnibus (GEO) database under accession number GSE275555., and the data can be accessed at the following link: https://www.ncbi.nlm.nih.gov/geo/query/acc.cgi?acc = GSE275555. Additionally, the original contributions presented in this study are included in the Supplementary Material of the article.

## Abbreviations

CBD: Congenital biliary dilatation
DMR: Differentially methylated region
CT: Computed tomography
MRI: Magnetic resonance imaging
SBP: Systolic blood pressure
DBP: Diastolic blood pressure
TG: Triglyceride
TC: Total cholesterol
ALT: Alanine aminotransferase
TBil: Total bilirubin
GGT: γ-Glutamyl transferase
CEBPE: CCAAT/enhancer-binding protein epsilon
DUSP9: Dual specificity protein phosphatase 9
POF1B: Actin binding protein1 B
OTUD5: OTU domain-containing protein 5
WDR45: WD repeat domain phosphoinositide-interacting protein 4
XIAP: X-linked inhibitor of apoptosis
