# Supplementary figures and images for: Genomic DNA methylation profile in peripheral blood of children with congenital biliary dilatation
Source: BMC Med Genomics. 2025 Oct 23;18:168. doi: 10.1186/s12920-025-02223-3 (PMC12548212; doi:10.1186/s12920-025-02223-3)

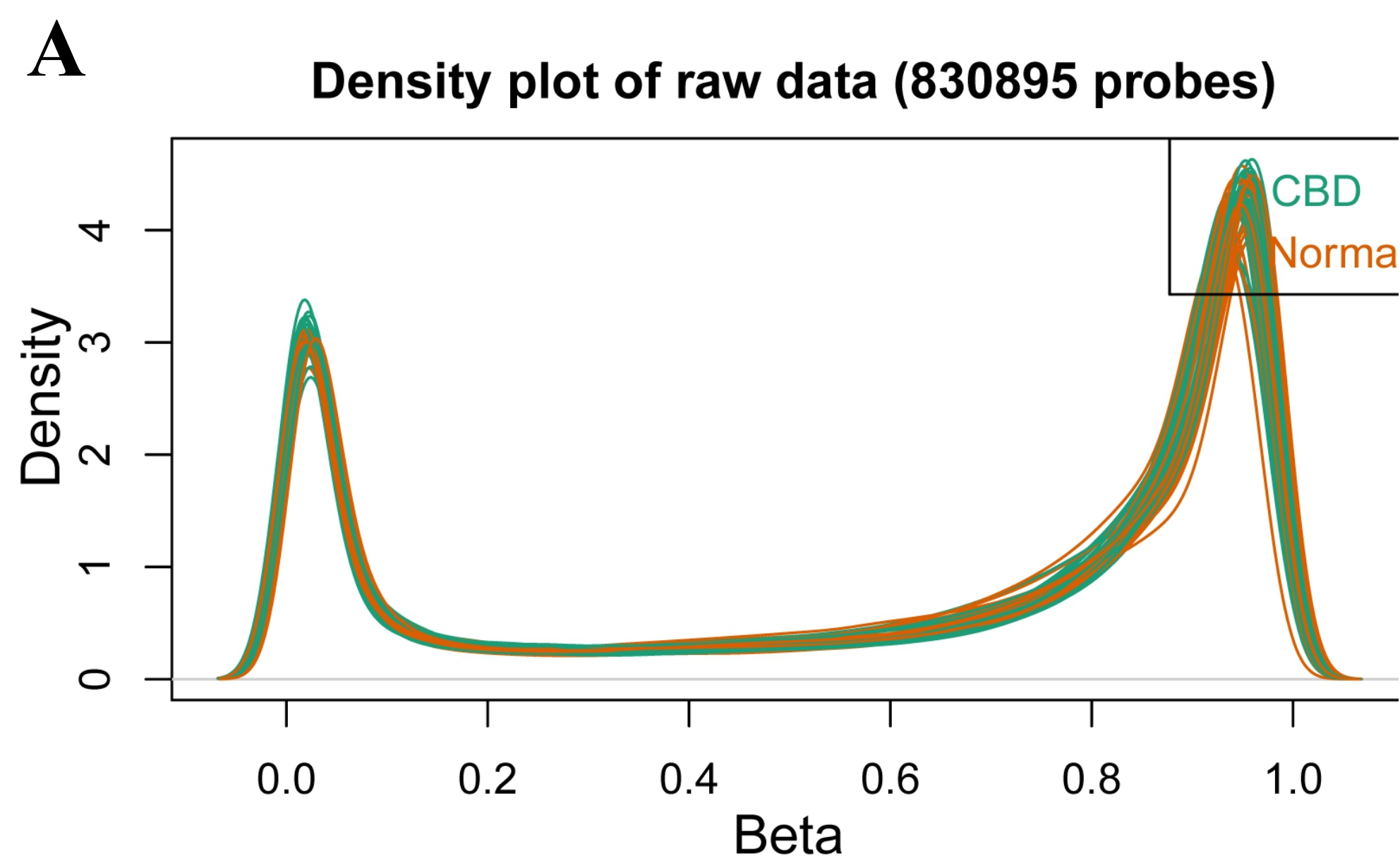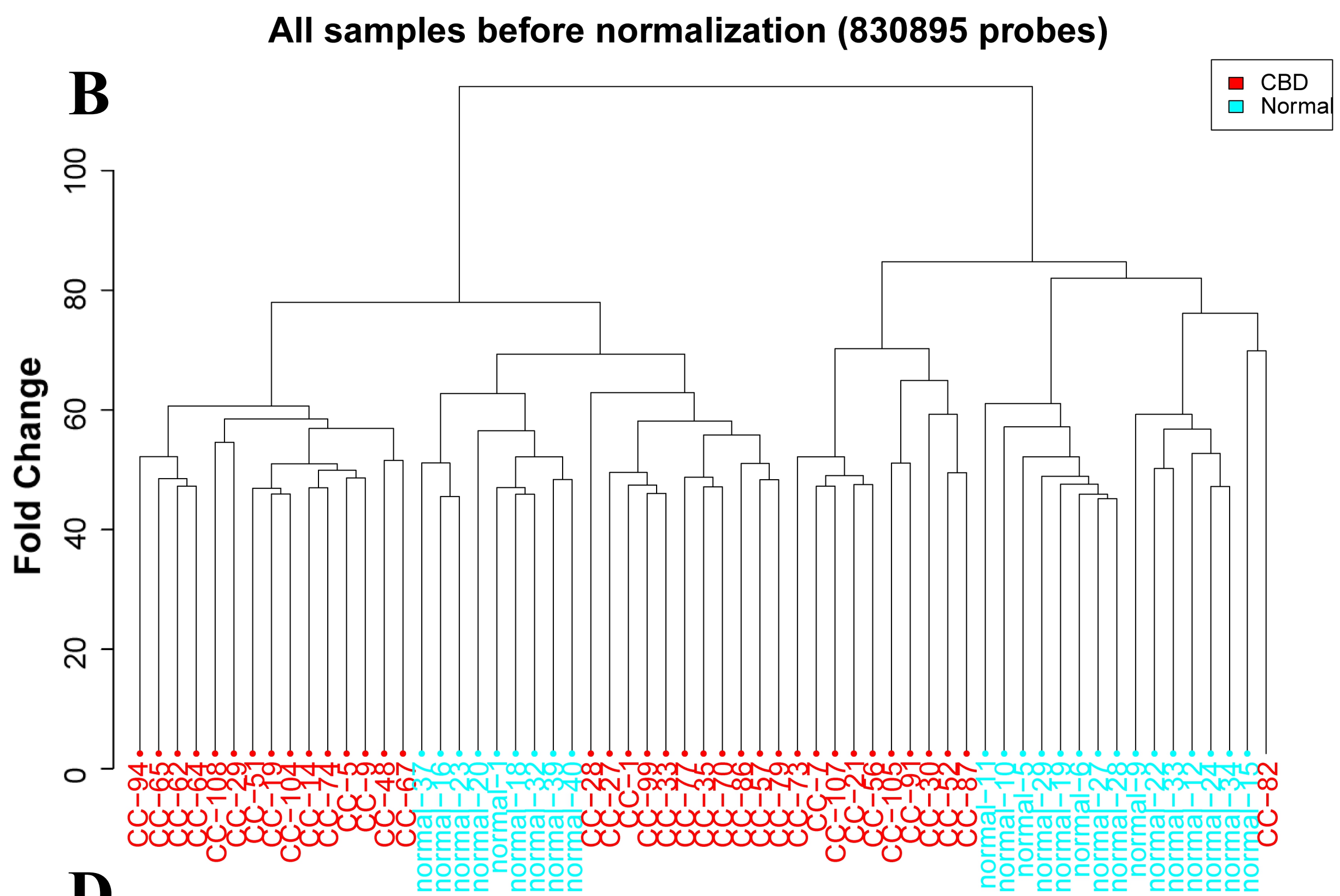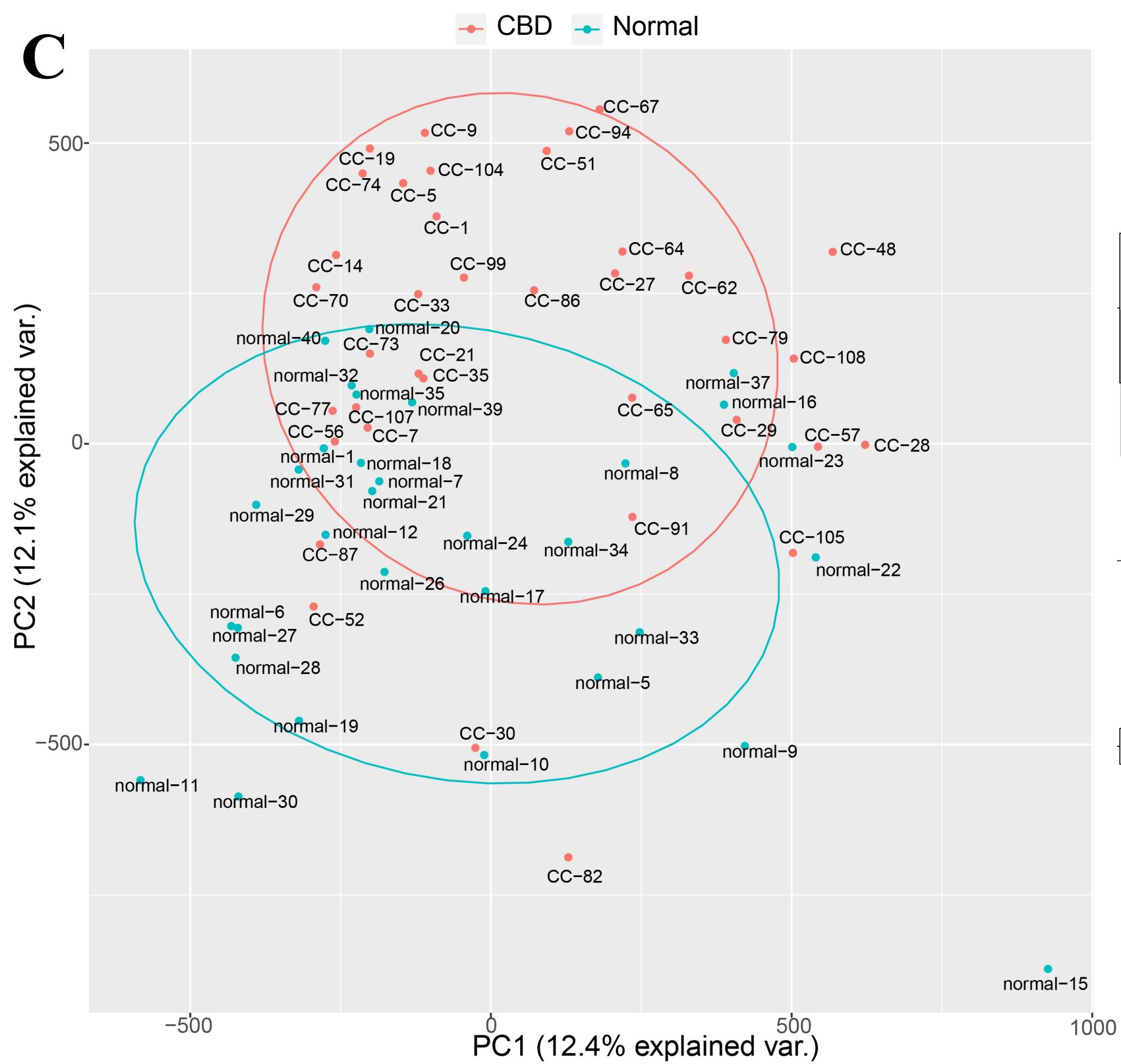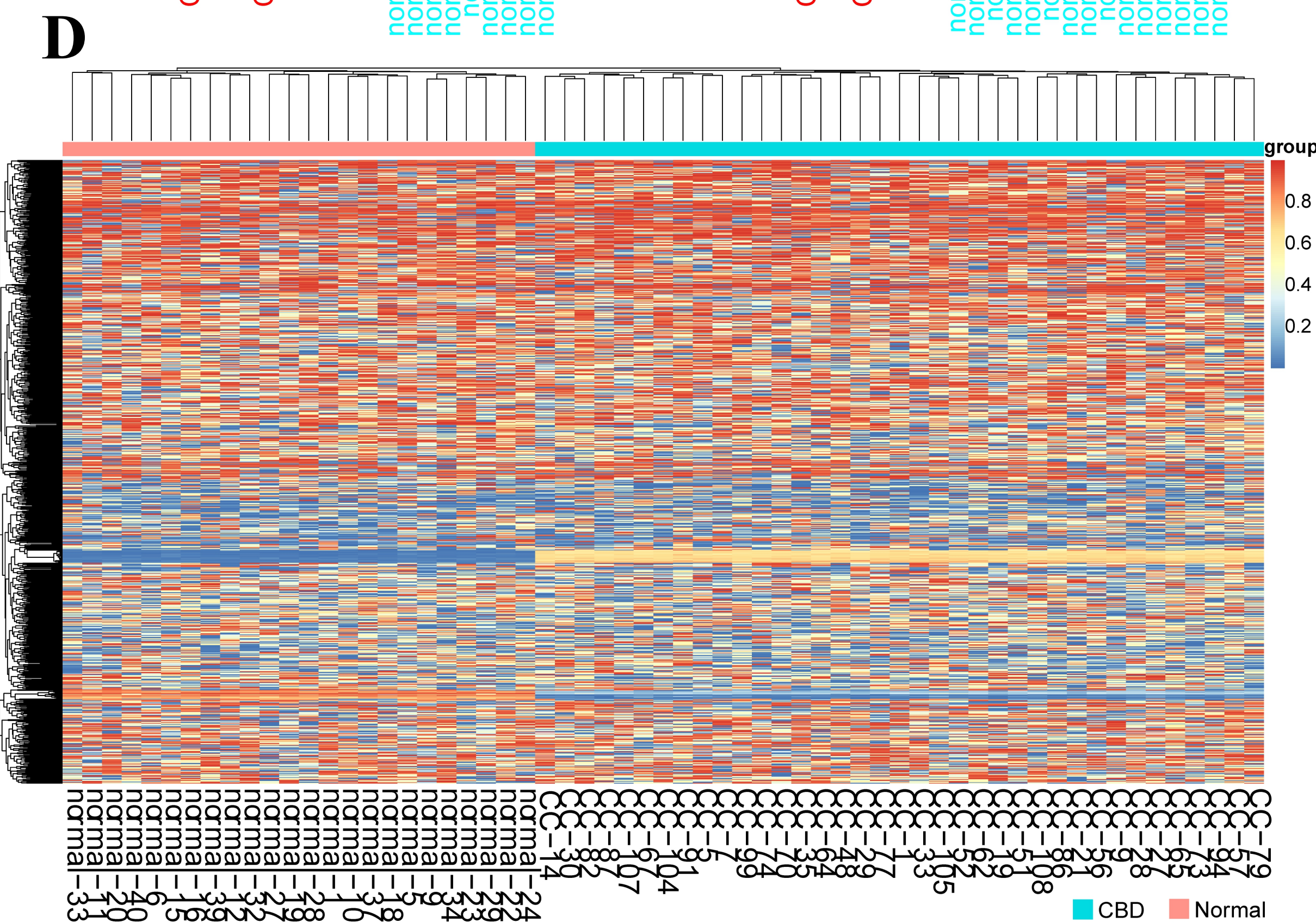

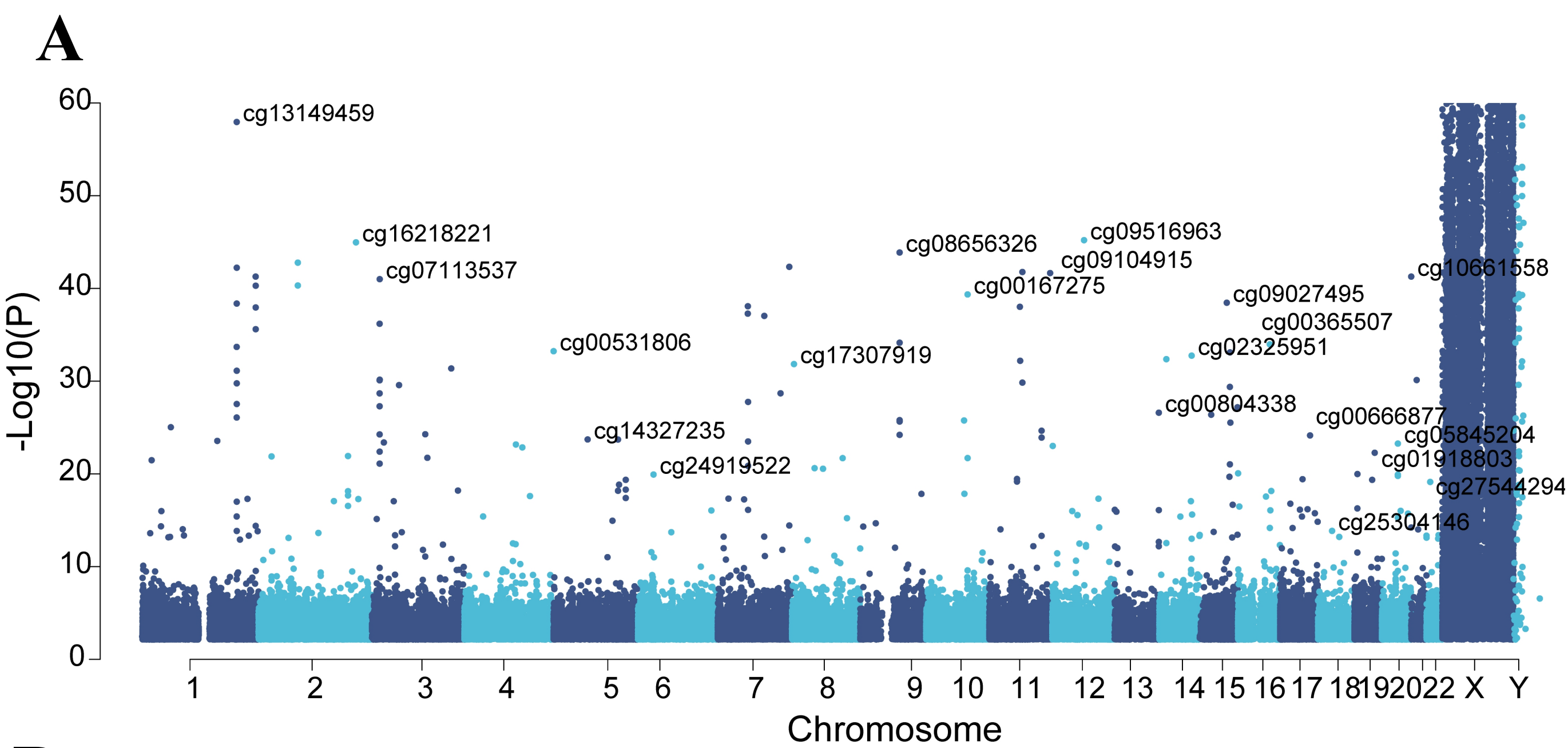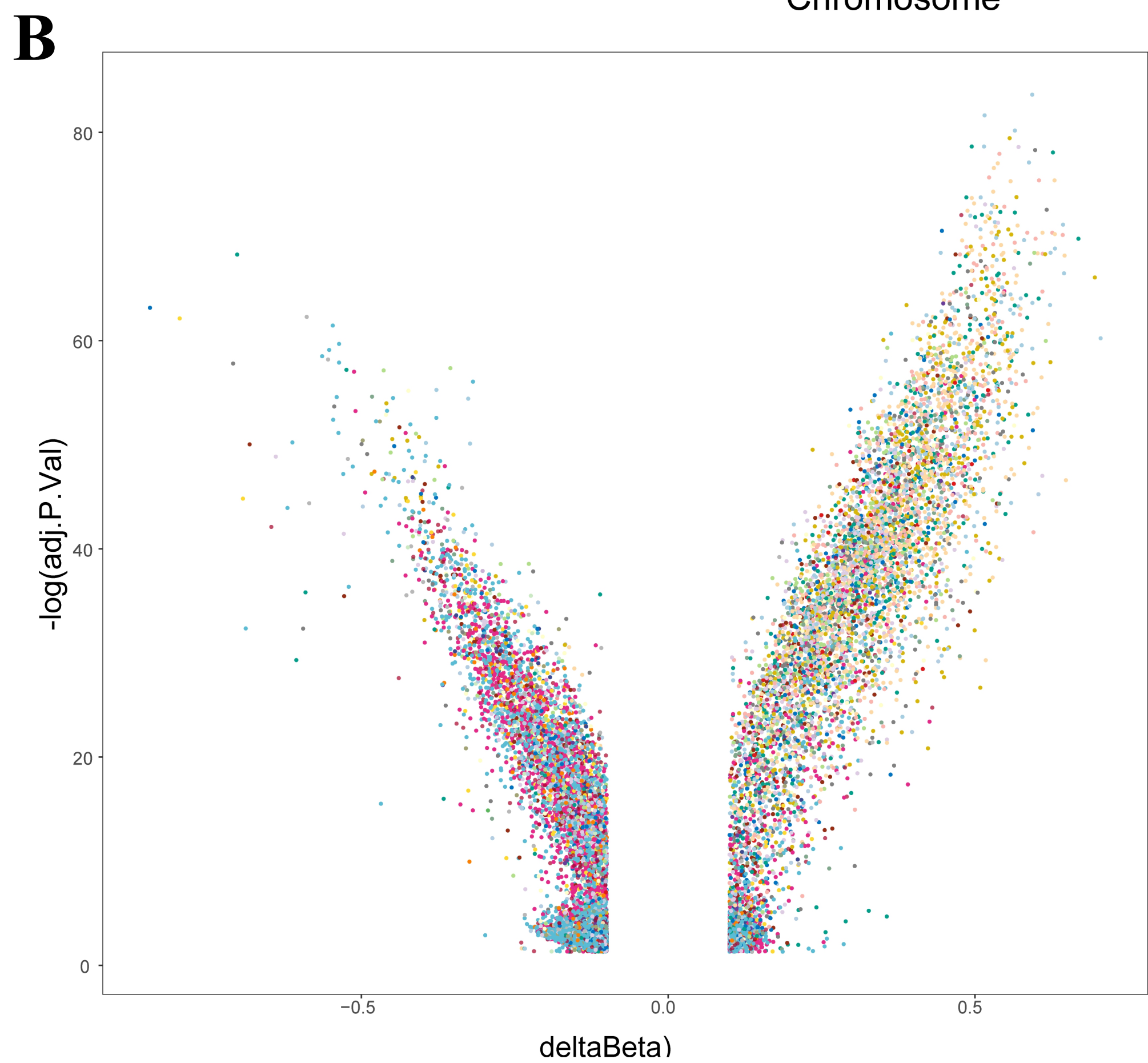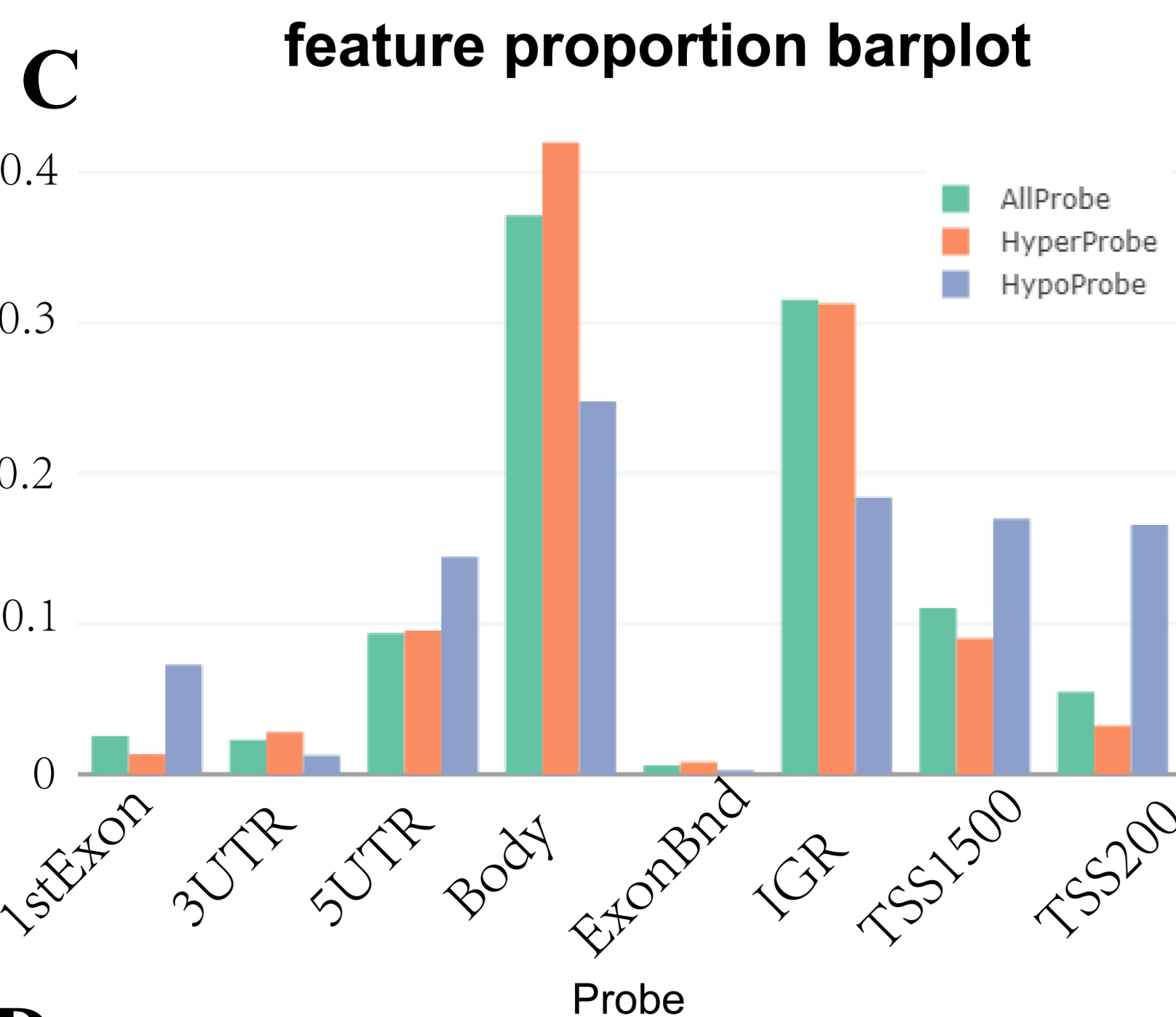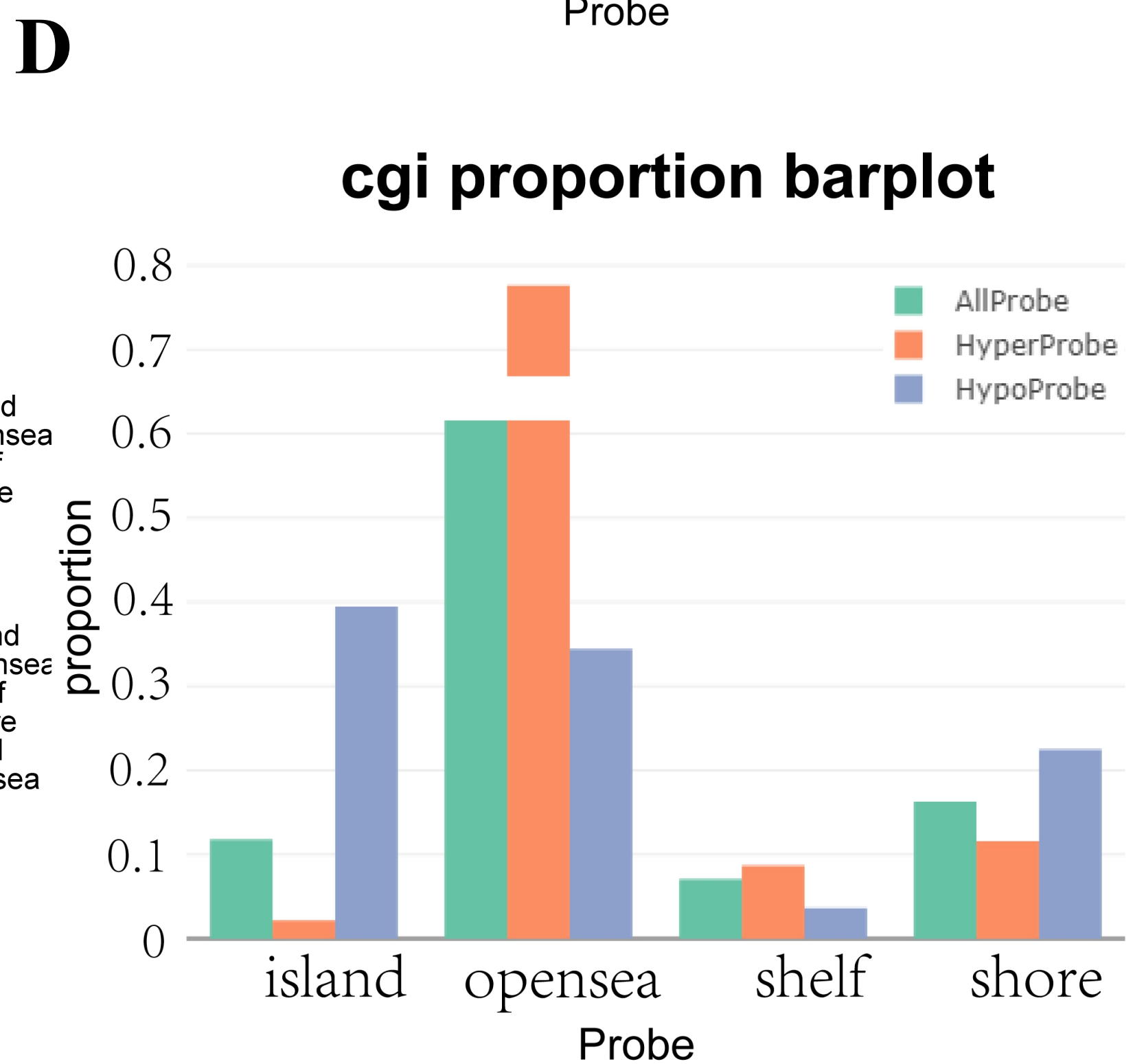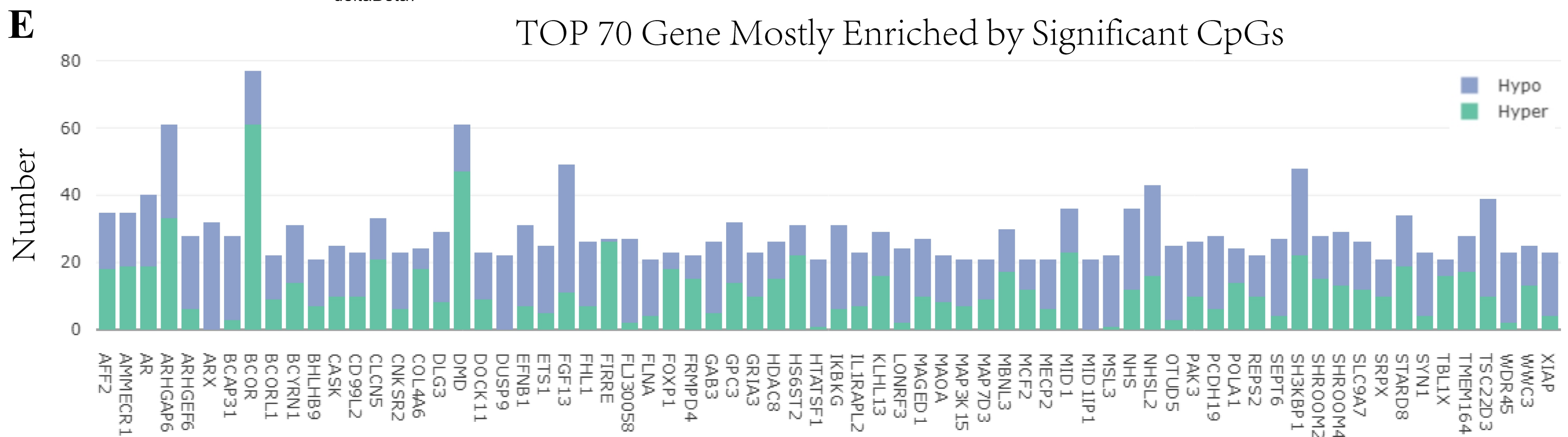

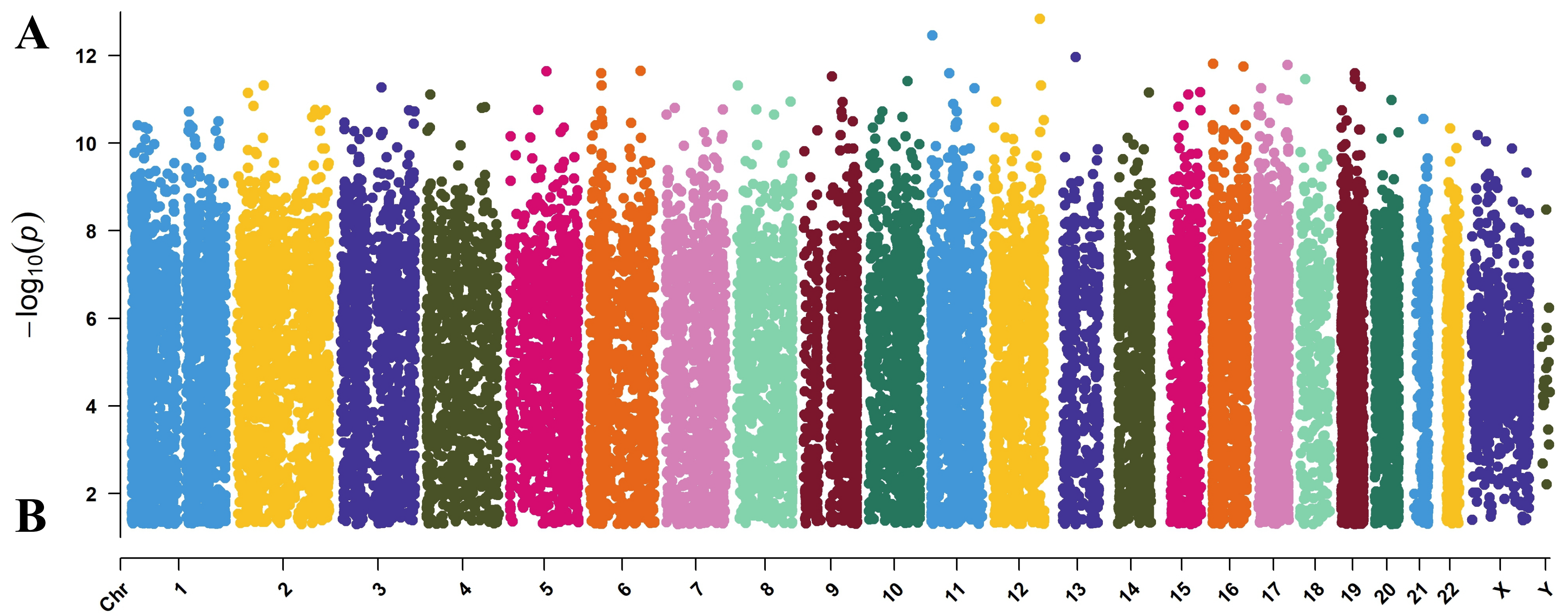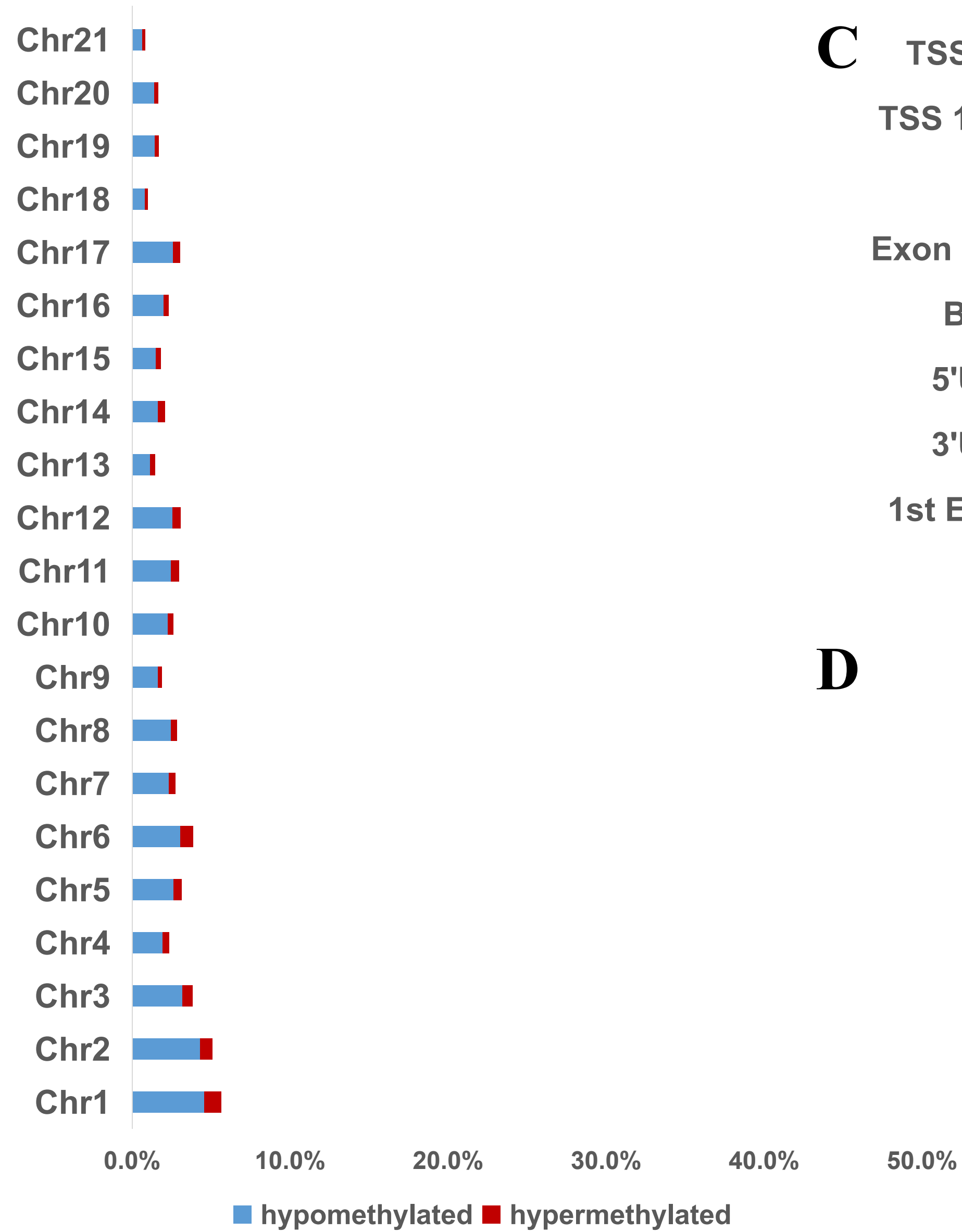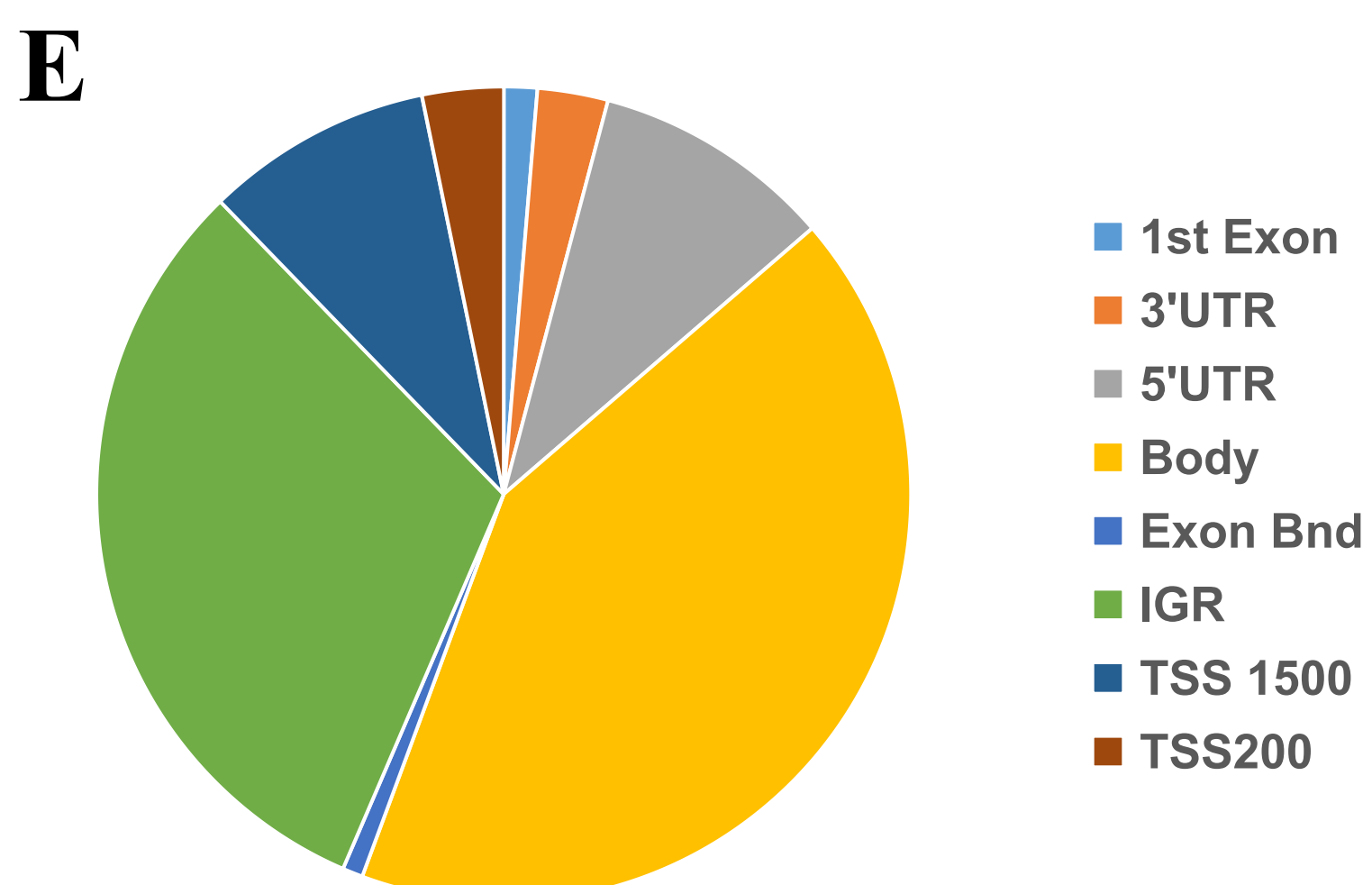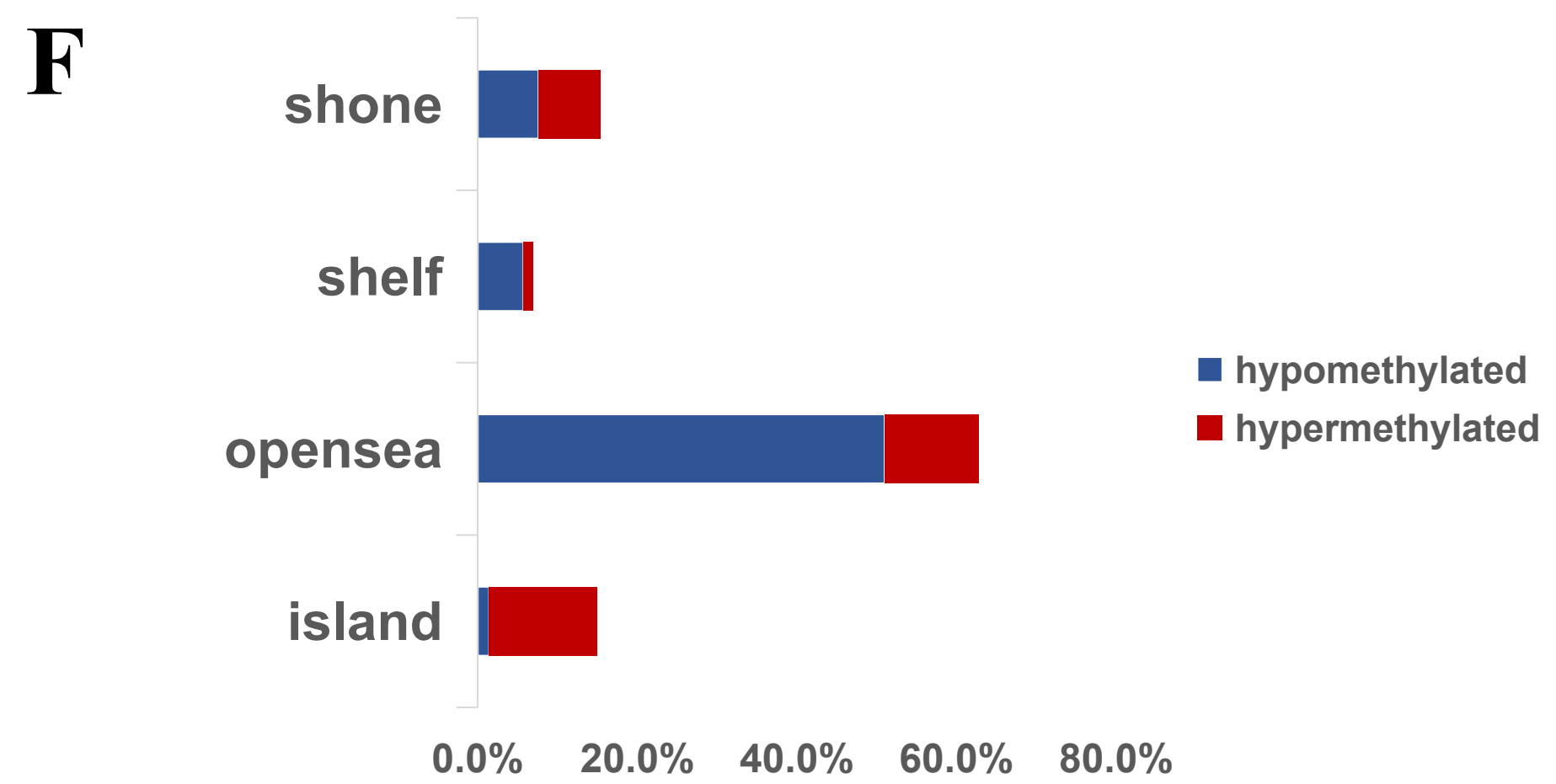

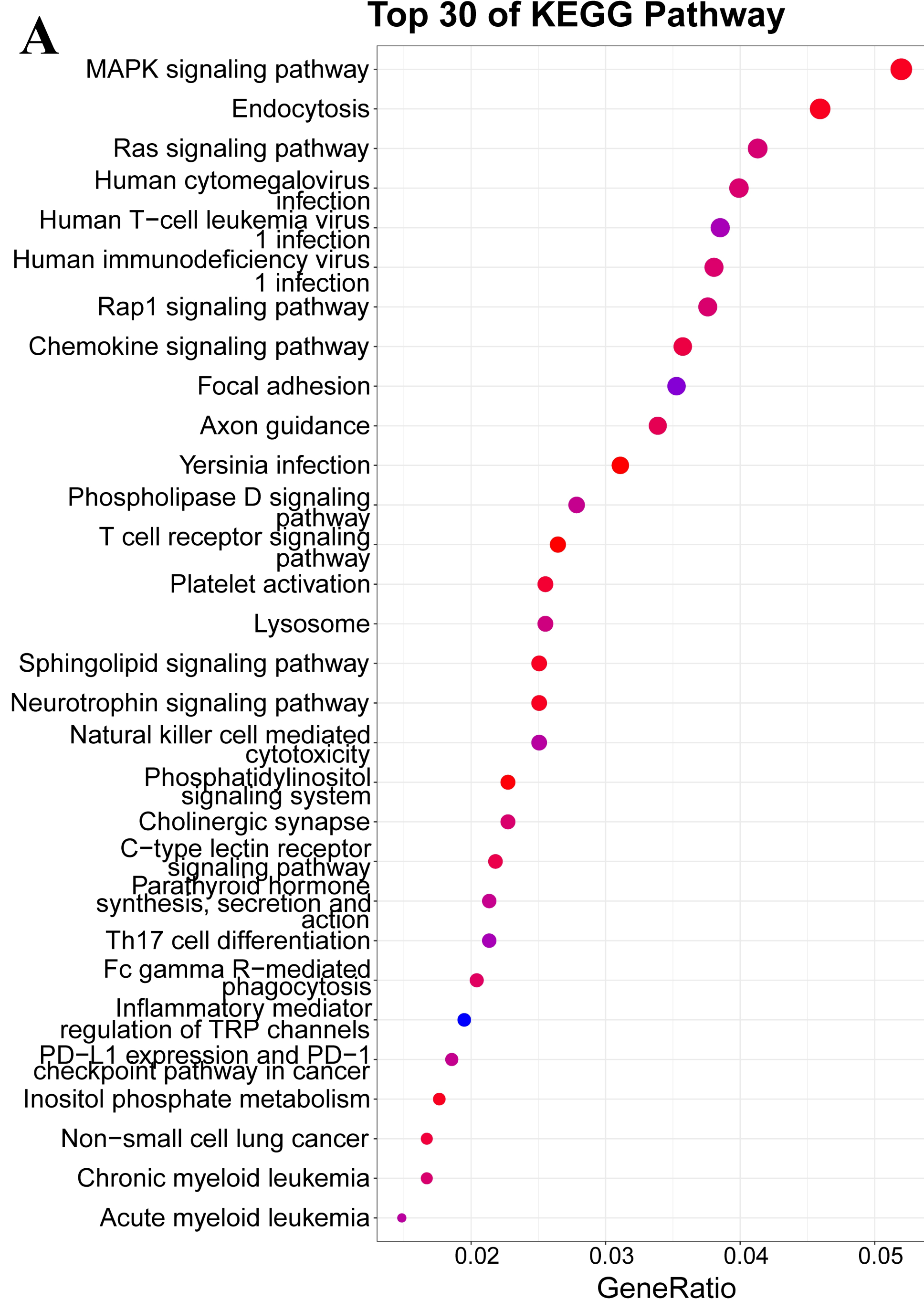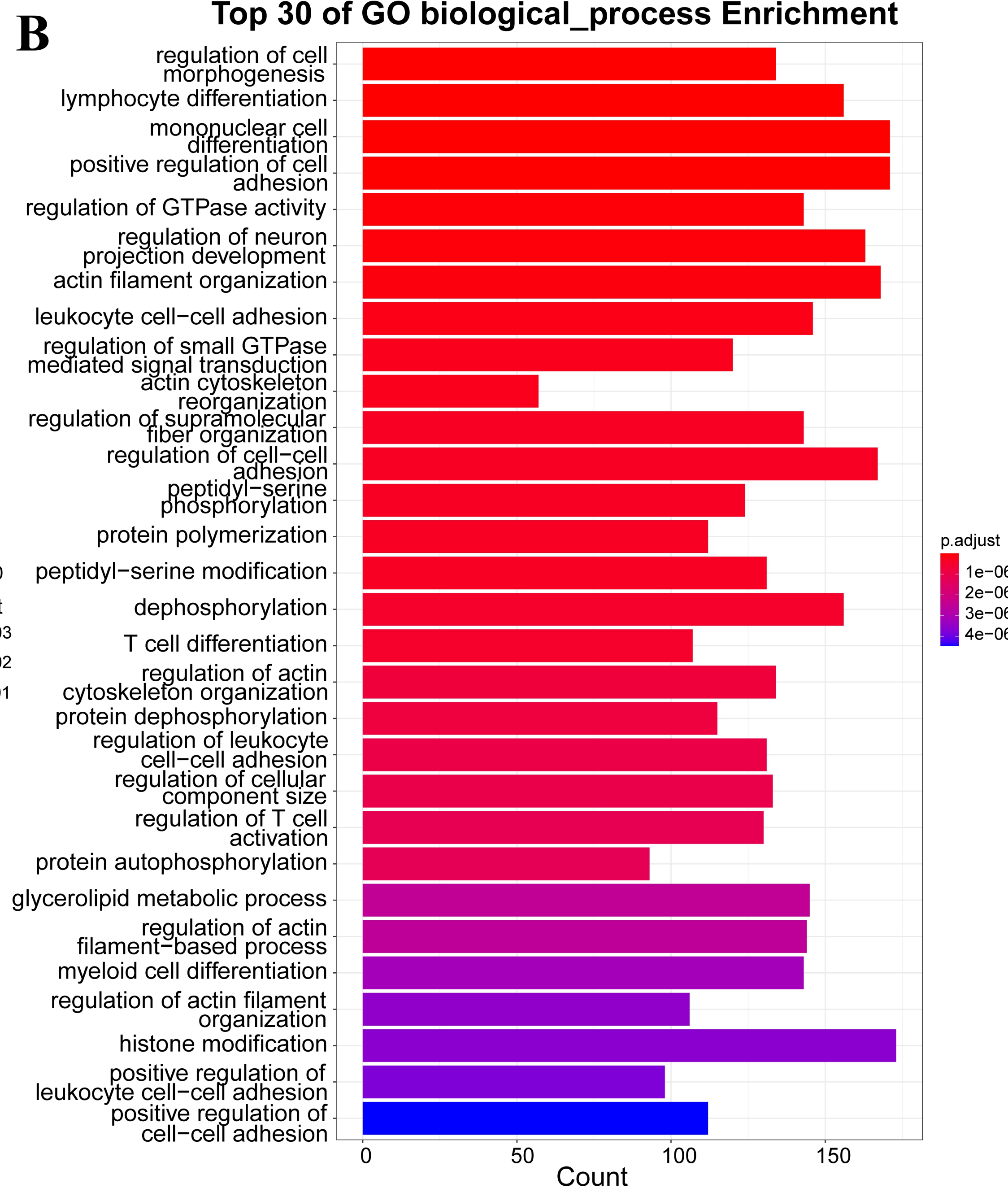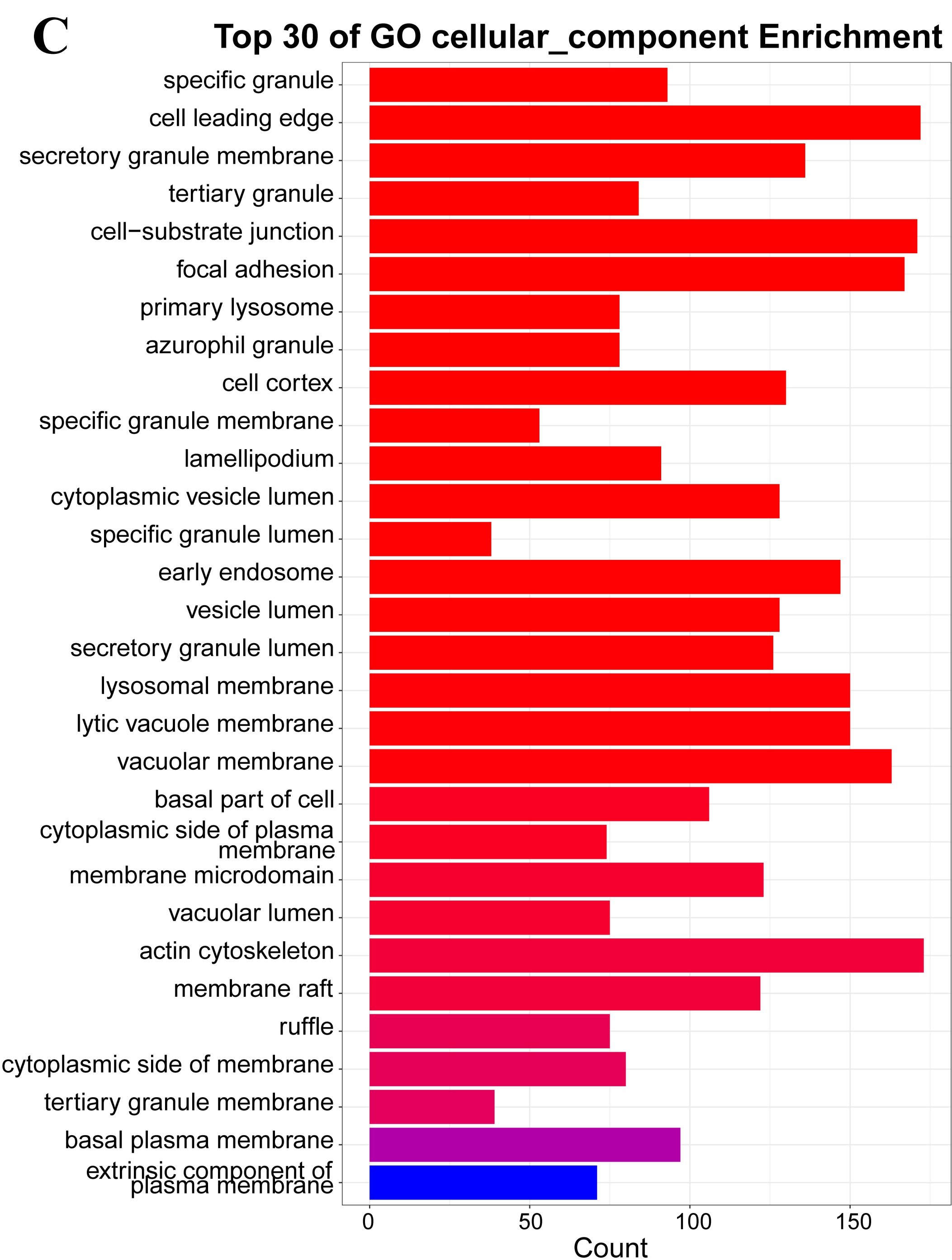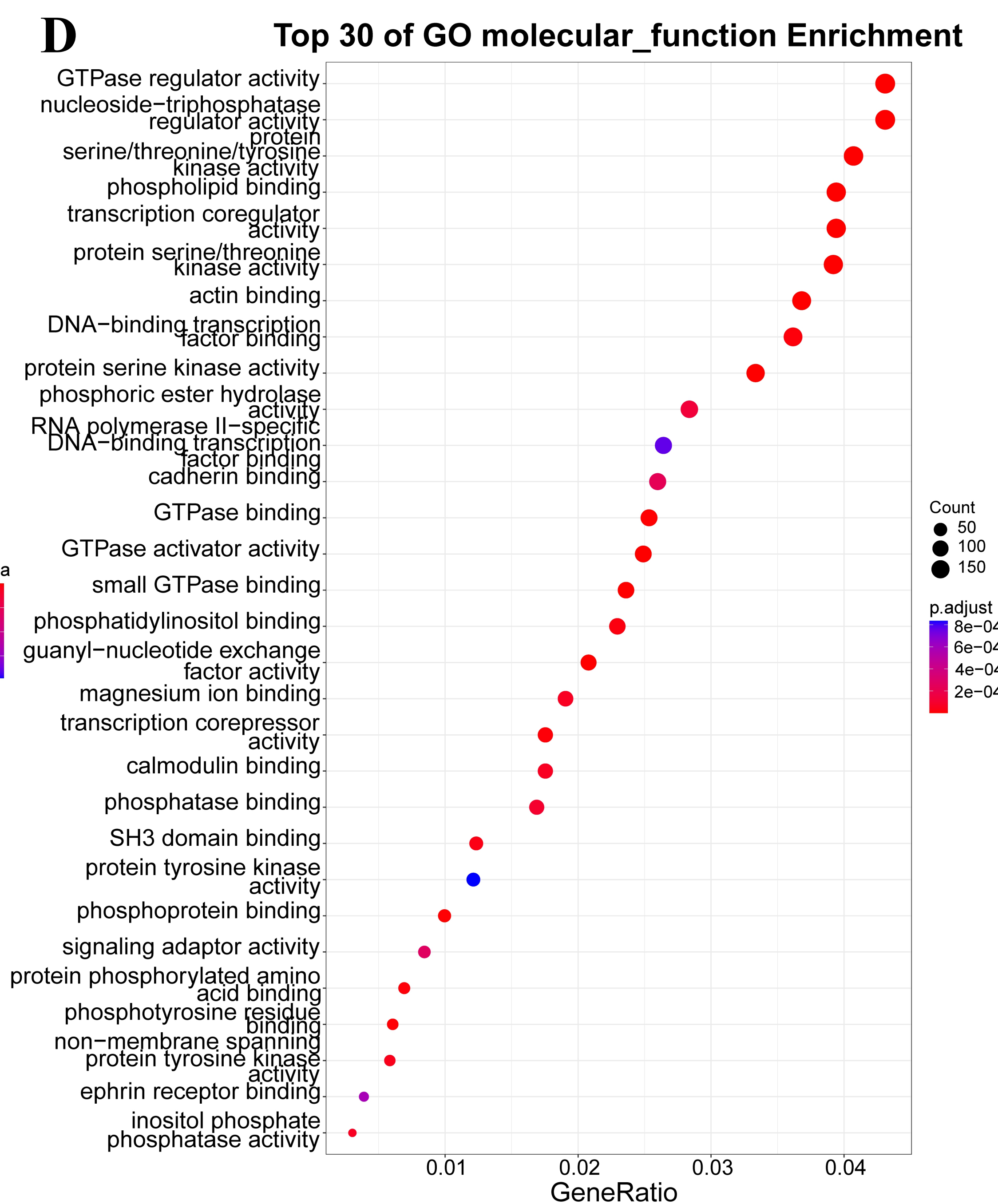

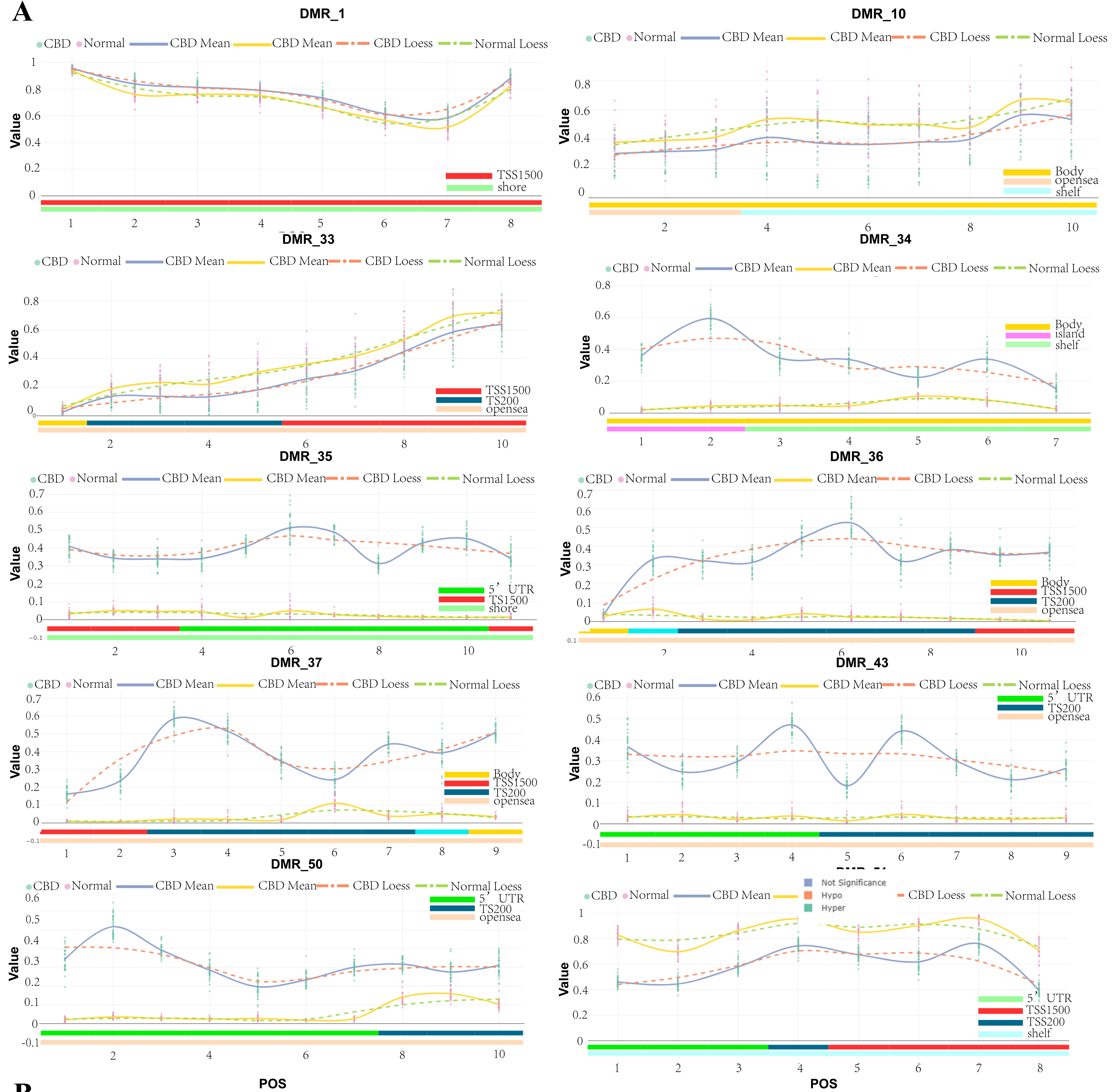

**A**

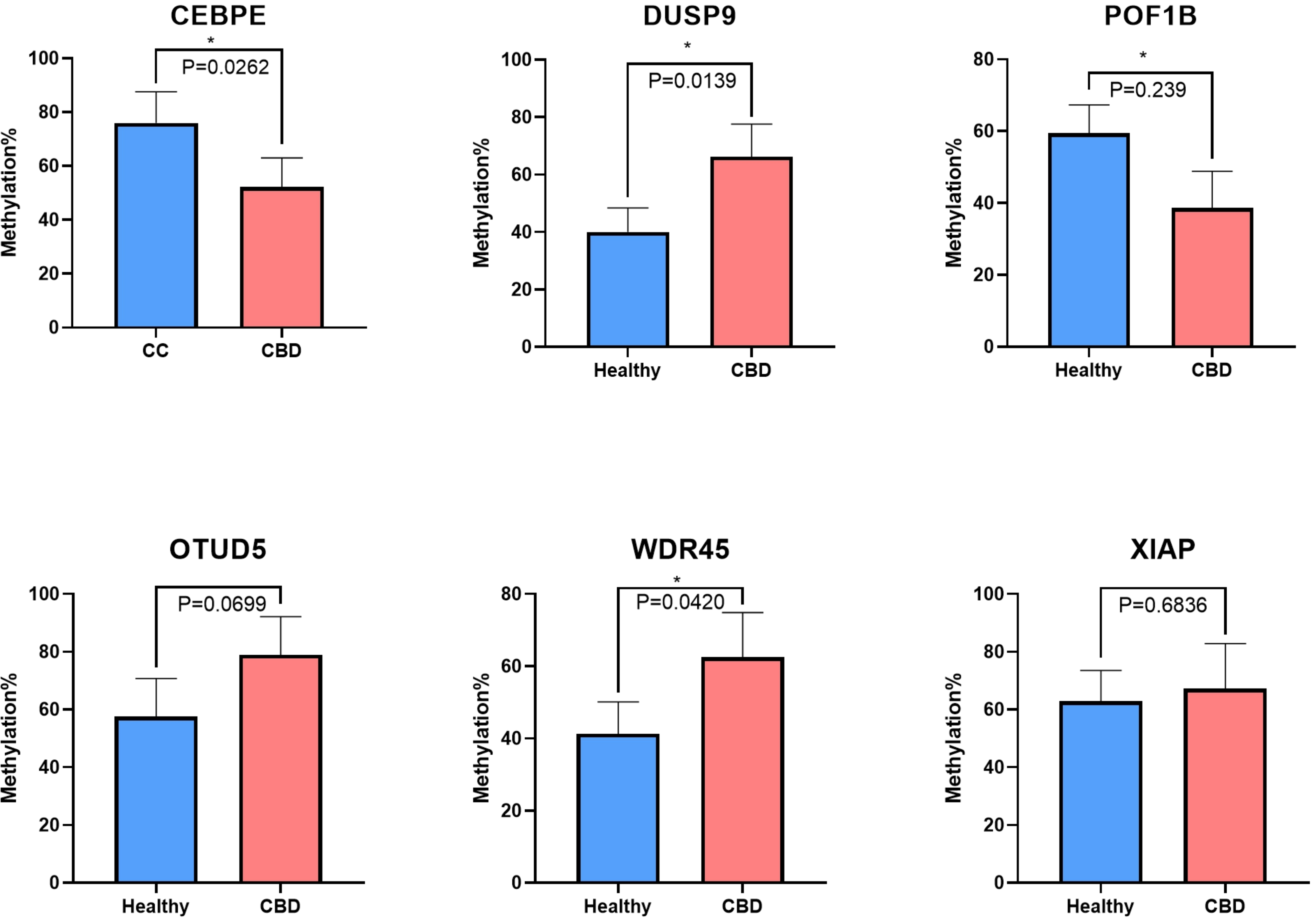

**B**

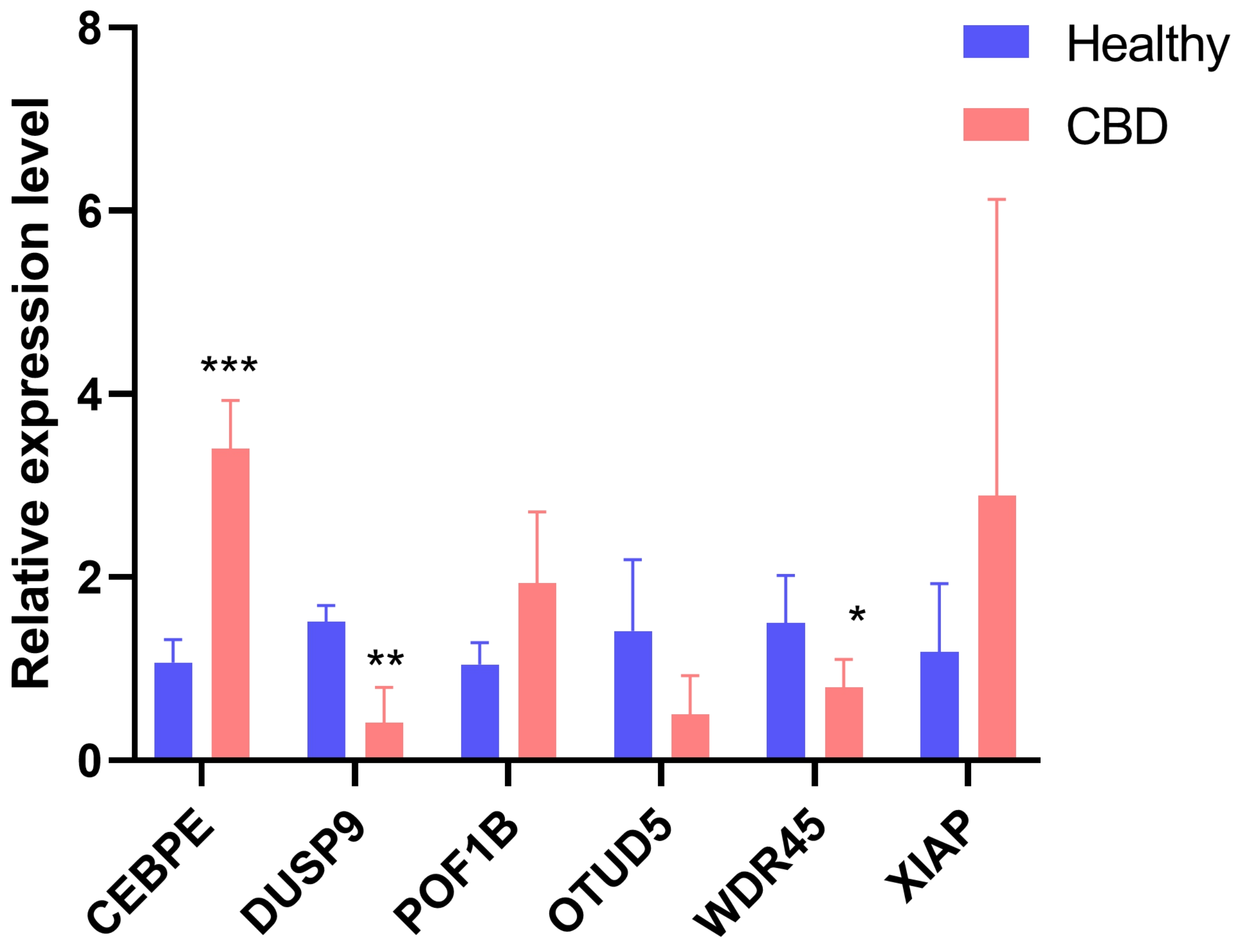

Supplement: Supplementary file 2 — Additional file 2 [file 12920_2025_2223_MOESM2_ESM.pdf]
